# Gut Microbiota Ecology and Inferred Functions in Children With ASD Compared to Neurotypical Subjects

**DOI:** 10.3389/fmicb.2022.871086

**Published:** 2022-06-09

**Authors:** Pamela Vernocchi, Maria Vittoria Ristori, Silvia Guerrera, Valerio Guarrasi, Federica Conte, Alessandra Russo, Elisabetta Lupi, Sami Albitar-Nehme, Simone Gardini, Paola Paci, Gianluca Ianiro, Stefano Vicari, Antonio Gasbarrini, Lorenza Putignani

**Affiliations:** ^1^Multimodal Laboratory Medicine Research Area, Unit of Human Microbiome, Bambino Gesù Children’s Hospital, Scientific Institute for Research, Hospitalization and Healthcare, Rome, Italy; ^2^Child and Adolescent Neuropsychiatry Unit, Department of Neuroscience, Bambino Gesù Children’s Hospital, Scientific Institute for Research, Hospitalization and Healthcare, Rome, Italy; ^3^GenomeUp, Rome, Italy; ^4^Institute for Systems Analysis and Computer Science “Antonio Ruberti,” National Research Council, Rome, Italy; ^5^Department of Diagnostics and Laboratory Medicine, Unit of Microbiology and Diagnostic Immunology, Unit of Microbiomics, Bambino Gesù Children’s Hospital, Scientific Institute for Research, Hospitalization and Healthcare, Rome, Italy; ^6^Department of Diagnostic and Laboratory Medicine, Unit of Microbiology and Diagnostic Immunology, Bambino Gesù Children’s Hospital, Scientific Institute for Research, Hospitalization and Healthcare, Rome, Italy; ^7^Department of Computer, Control and Management Engineering, Sapienza University of Rome, Rome, Italy; ^8^CEMAD Digestive Disease Center, Fondazione Policlinico Universitario “A. Gemelli” Scientific Institute for Research, Hospitalization and Healthcare, Università Cattolica del Sacro Cuore, Rome, Italy; ^9^Department of Diagnostics and Laboratory Medicine, Unit of Microbiology and Diagnostic Immunology, Unit of Microbiomics, and Multimodal Laboratory Medicine Research Area, Unit of Human Microbiome, Bambino Gesù Children’s Hospital, Scientific Institute for Research, Hospitalization and Healthcare, Rome, Italy

**Keywords:** autism spectrum disorders, gastrointestinal symptoms, gut microbiota ecology, fecal secretory IgA, zonulin, lysozyme, microbial and KEGG biomarkers

## Abstract

Autism spectrum disorders (ASDs) is a multifactorial neurodevelopmental disorder. The communication between the gastrointestinal (GI) tract and the central nervous system seems driven by gut microbiota (GM). Herein, we provide GM profiling, considering GI functional symptoms, neurological impairment, and dietary habits. Forty-one and 35 fecal samples collected from ASD and neurotypical children (CTRLs), respectively, (age range, 3–15 years) were analyzed by 16S targeted-metagenomics (the V3–V4 region) and inflammation and permeability markers (i.e., sIgA, zonulin lysozyme), and then correlated with subjects’ metadata. Our ASD cohort was characterized as follows: 30/41 (73%) with GI functional symptoms; 24/41 (58%) picky eaters (PEs), with one or more dietary needs, including 10/41 (24%) with food selectivity (FS); 36/41 (88%) presenting high and medium autism severity symptoms (HMASSs). Among the cohort with GI symptoms, 28/30 (93%) showed HMASSs, 17/30 (57%) were picky eaters and only 8/30 (27%) with food selectivity. The remaining 11/41 (27%) ASDs without GI symptoms that were characterized by HMASS for 8/11 (72%) and 7/11 (63%) were picky eaters. GM ecology was investigated for the overall ASD cohort versus CTRLs; ASDs with GI and without GI, respectively, versus CTRLs; ASD with GI versus ASD without GI; ASDs with HMASS versus low ASSs; PEs versus no-PEs; and FS versus absence of FS. In particular, the GM of ASDs, compared to CTRLs, was characterized by the increase of Proteobacteria, Bacteroidetes, Rikenellaceae, Pasteurellaceae, *Klebsiella, Bacteroides*, *Roseburia*, *Lactobacillus*, *Prevotella*, *Sutterella*, *Staphylococcus*, and *Haemophilus*. Moreover, *Sutterella*, *Roseburia* and *Fusobacterium* were associated to ASD with GI symptoms compared to CTRLs. Interestingly, ASD with GI symptoms showed higher value of zonulin and lower levels of lysozyme, which were also characterized by differentially expressed predicted functional pathways. Multiple machine learning models classified correctly 80% overall ASDs, compared with CTRLs, based on *Bacteroides*, *Lactobacillus*, *Prevotella*, *Staphylococcus*, *Sutterella*, and *Haemophilus* features. In conclusion, in our patient cohort, regardless of the evaluation of many factors potentially modulating the GM profile, the major phenotypic determinant affecting the GM was represented by GI hallmarks and patients’ age.

## Introduction

Autism spectrum disorder (ASD) is a multifactorial neurodevelopmental disorder, which is characterized by deficits in social interactions, communication, and by the presence of restrictive and repetitive behaviors, interests, and activities (American Psychiatric Association [APA], 2013). In United States, across all 11 Autism and Developmental Disabilities Monitoring (ADDM) Network sites, monitored by the Center for Disease Control and Prevention (CDCP), ASD prevalence was 18.5 per 1,000 (one in 54) children aged 8 years (Maenner et al., 2020), and European studies have reported prevalence of 1 and 2% in the childhood population (Carta et al., 2020). Differences in prevalence depend on methodological approaches, demographic factors, and geographical area (Lyall et al., 2017). However, ASD is an etiologically heterogeneous disorder involving multiple factors, such as genetic (Jonsson et al., 2014; Leblond et al., 2014; Wilkinson et al., 2015) and environmental factors (Herbert, 2010; Raz et al., 2015), including pre- and/or post-natal factors (de Theije et al., 2011).

Among ASD comorbidities, gastrointestinal (GI) symptoms are of particular interest, given their reported prevalence (Buie et al., 2010; Coury et al., 2012) and correlation with ASD severity (Adams et al., 2011; Chernikova et al., 2021). GI symptoms include abdominal pain, constipation, diarrhea, bloating, and gastroesophageal reflux (GERD) (Horvath and Perman, 2002; Valicenti-McDermott et al., 2006; Nikolov et al., 2009). Among neurological symptoms, irritability (Bresnahan et al., 2015), anxiety and affective disorders (Valicenti-McDermott et al., 2006; Fulceri et al., 2016), dysregulation and externalizing problems (Horvath and Perman, 2002), rigid/compulsive behaviors (Peters et al., 2014; Marler et al., 2017), increased sensory sensitivity (Mazurek et al., 2013), and sleep problems (Horvath and Perman, 2002; Maenner et al., 2012) have been reported in patients with ASD in presence/absence of GI symptoms (Adams et al., 2011; Wang et al., 2011; Gorrindo et al., 2012; Chaidez et al., 2014).

In the last decade, many studies have highlighted the role of the gut microbiota (GM) in neurodevelopmental disorders (Hsiao et al., 2013; Borre et al., 2014; Kelly et al., 2017), particularly in ASD (Parracho et al., 2005; Finegold et al., 2010; Adams et al., 2011; Williams et al., 2011, 2012; Kang et al., 2013). Indeed, the GM is known to influence social-behavior and brain physiology through a diverse set of pathways (Flannery et al., 2020; Buffington et al., 2021), including immune activation (Furness et al., 1999; Kruglov et al., 2013), production of microbial peptides, metabolites, and various neuromodulators and neurotransmitters (Sherwin et al., 2019). In fact, the gut is linked to different brain functions acting on emotional and cognitive brain regions, such as prefrontal cortex, limbic system, and hypothalamus (Davies et al., 2021). Therefore, the communication by cross-talk interaction between the central nervous system (CNS) and the GI tract, called “gut-brain axis,” plays a key role in neurological disease pathophysiology and seems apparently driven by GM ecology and function (Collins et al., 2012; Ristori et al., 2019).

Several studies have evidenced that the gut microbial composition of the ASD enterophenotype is characterized by an increase in harmful microbes and a decrease in beneficial ones (Adams et al., 2011; Wang et al., 2011; Liu S. et al., 2019). Particularly, few studies based on targeted-metagenomics (Liu S. et al., 2019; Ma et al., 2019; Berding and Donovan, 2020) have highlighted specific overrepresented ASD-related microbial signatures, such as *Ruminococcus*, *Sutterella*, *Enterococcus*, *Prevotella*, and *Fecalibacterium prausnitzii* (Kang et al., 2013; De Angelis et al., 2015; Tomova et al., 2015; Fung et al., 2017).

However, GM also plays a critical role in maintaining the intestinal barrier integrity, protecting from bacterial toxins’ passage into the bloodstream, a phenomenon related to peripheral inflammation and induction of behavioral alterations and damage of a blood-brain barrier, as demonstrated in animal models (Stolp et al., 2011; Liu F. et al., 2019).

Thus, changes in intestinal permeability due to tight junctions’ modulation may reflect low GM functionality, as well as low antibacterial defense. Zonulin is a well-known biomarker of permeability, usually related to chronic diseases, such as diabetes, celiac disease, inflammatory bowel disease, or obesity (Fasano, 2012; Sturgeon and Fasano, 2016), but also to ASD-associated comorbidities (Esnafoglu et al., 2017; Fasano and Hill, 2017). Secretory IgAs (sIgAs) represent the first immune barrier of defense in the gut protecting the intestinal epithelium from pathogens and enteric toxins (Mantis et al., 2011), hence mediating the innate immune defense mechanism at the level of the gut mucosa, as also reported in patients with ASD (Zhou et al., 2018). Fecal lysozyme is an alkaline glycosidase secreted by granulocytes, macrophages, Paneth cells, Brunner’s glands, and normal colonic crypt cells, playing antimicrobial action by hydrolyzation of specific glycosidic bonds of mucopolysaccharides of Gram-positive bacteria wall.

Elevated levels of fecal lysozyme have been identified in colonic IBD when compared to healthy controls (Klass and Neale, 1978; Veer et al., 1998) but also in patients with ASD (Adams et al., 2011).

Herein, we considered an ecological and inferred functional description of the GM based on GI and severity symptoms, dietary habits, and fecal host biomarkers, underlying GI hallmarks and age as the main driving features of the GM profile in our ASD cohort.

## Materials and Methods

### Patient Characteristics and Sample Collection

Forty-one patients with ASD aged 3–15 years (average age, 6.5 years, SD ± 3.42), of whom 36 males and 5 females, with a male gender frequency of 88%, who were enrolled, were recruited at the Bambino Gesù Children’s Hospital (Rome, Italy) and University Hospital Agostino Gemelli (Rome, Italy). Thirty-five aged-matched neurotypical children (average age, 8 years, SD ± 3.62; 21 males and 14 females) were enrolled during an epidemiological survey carried out at the Human Microbiome Unit of Bambino Gesù Children’s Hospital in Rome (BBMRI Human Micro-biome Biobank, OPBG) to generate a reference digital biobank of neurotypical healthy subjects (controls, CTRLs).

Autism spectrum disorder inclusion criteria were: age, 3–18 years; diagnosis of ASD according to criteria of the Diagnostic and Statistical Manual of Mental Disorders, fifth edition (DSM-5); confirmation by the Autism Diagnostic Observation Schedule, second edition (ADOS-2) and, when available, by the Autism Diagnostic Interview—Revised (ADI-R). The characterization of ASD core symptoms, cognitive level, and behavioral problems was conducted by trained clinical psychologists and psychiatrists. All the patients who did not meet the inclusion criteria and did not have a diagnosis of ASD were excluded.

**FIGURE 1:**
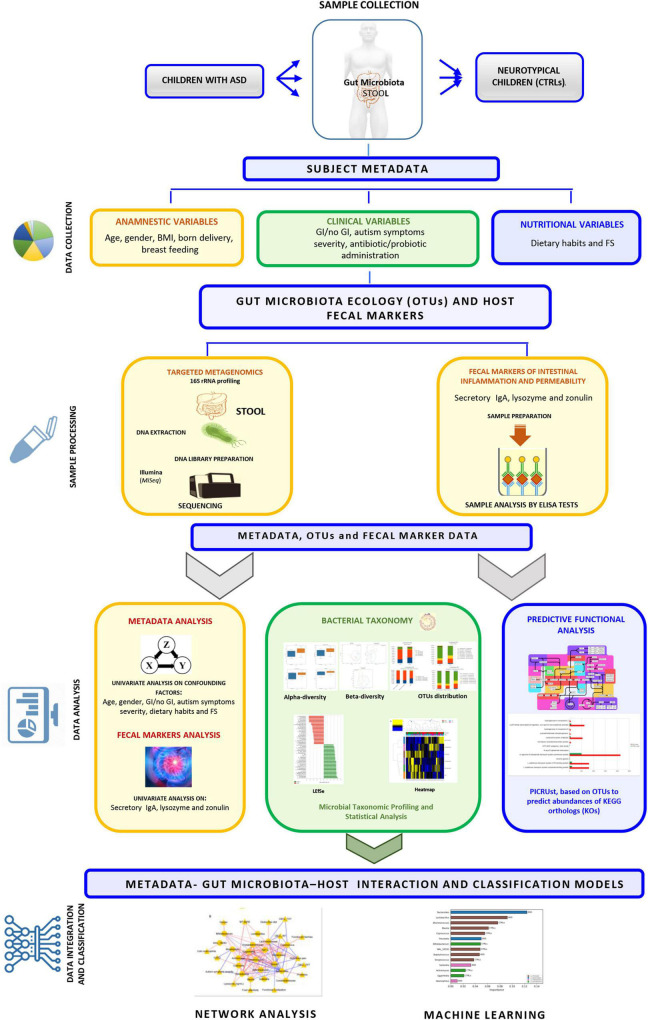
Graphical representation of the experimental workflow performed in the study.

The participants’ metadata included clinical, anamnestic, and nutritional data [i.e., presence of food selectivity (FS) as: elimination diet, gluten free or casein free], probiotics’ supplementation and antibiotics’ administration (Supplementary Table 1 and Figure 1). Among clinical parameters (i.e., weight, height) for body mass index (BMI) calculation, the percentiles of body mass index (pBMI) were evaluated according to Center for Disease Control and Prevention (CDCP) growth charts for children and teens ages 2–19 years^1^ (Supplementary Table 1, sheet General Characteristics and Nutritional info and Figure 1). Fecal samples were collected at the reference node of the Human Microbiome Biobanking and Biomolecular Resources Research Infrastructure (BBMRI) of the OPBG Human Microbiome Unit and stored at −80°C until processing.

#### Gastrointestinal Assessment

A questionnaire based on Rome IV classification (Drossman and Hasler, 2016) was exploited to evaluate GI symptoms, such as the presence of abdominal pain, functional diarrhea, constipation, and vomiting disorders. Also, presence or absence of GERD (Wasilewska and Klukowski, 2015) and colonic eosinophilia (Katzka, 2014) from intestinal biopsies was considered when available. Hence, the patients with ASD were stratified into subgroups based on the presence or absence of one or more GI disorders to identify possible relationships with GM differences based on ASD GI-related enterophenotypes (Supplementary Table 1 sheet GI features and Figure 1). Finally, the two groups, namely, with and without GI symptoms, were considered for differential GM ecology description.

#### Neuropsychological Assessment

The cognitive level (intelligent quotient, IQ; and developmental quotient, DQ) was assessed according to age, language level, compliance, and timing of IQ evaluation by WISC-IV (Lang et al., 2015), non-verbal IQ by Leiter-3 (Roid et al., 2013) or general quotient (GQ) by Griffiths Mental Development Scales Extended, revised for ages 2–8 (Sannio Fancello et al., 2007).

Autism symptoms were evaluated using ADOS-2 (Lord et al., 2012), a semi-structured direct assessment of communication, social interaction, play or imaginative use of materials for individuals with a suspected diagnosis of ASD, according to the language level and ranging from non-verbal to verbal fluency. The ADOS-2 was administered and scored by licensed clinicians with a demonstrated clinical capacity of its usage. For some of the patients, the diagnosis was also confirmed by ADI-R (Lord et al., 1994), a parent semi-structured interview. To assess the severity of ASD symptomatology, total raw scores were calibrated and transformed into calibrated severity score (CSS) (Gotham et al., 2009), with a score ranging from 1 to 10. Based on the CSS, the patients were divided into three levels of severity according to ADOS scores criteria (American Psychiatric Association [APA], 2013). Behavioral and psychopathological problems were assessed by the Achenbach System of Empirically Based Assessment (ASEBA) questionnaire (Rescorla, 2005), specifically by using the 1.5–5 years’ Child Behavior Checklist (CBCL) and the 6–18 years’ CBCL questionnaires, reported by parents. The parents evaluated their children’s behavior during the preceding 6 months on a 3-point Likert scale for each item (0 = not true; 1 = somewhat or sometimes true; 2 = very true or often true). Neuropsychologists obtained scores from Internalizing, Externalizing, and Total Problems Scales. For 1.5–5 CBCL, the internalizing domain was based on four syndrome scales: emotionally reactive, anxious and depressed, somatic complaining, and withdrawn. According to the cut-off thresholds of Achenbach et al. (2003), Internalizing, Externalizing, and Total Problems’ scales were classified according to symptoms’ t-scores: clinically relevant (≥65), borderline (60–64), and non-clinical (<60) (Supplementary Table 1, sheet Neuropsycological features and Figure 1).

### Ethics Statement

The study was approved by the OPBG Ethics Committees for both patients with ASD and healthy subjects’ recruitment, respectively, 1404_OPBG_2017 and 1113_OPBG_2016 protocols. The project was conducted in accordance with the Principles of Good Clinical Practice and the Declaration of Helsinki. Written informed consent was obtained from all the participants.

### Fecal Markers of Intestinal Inflammation and Permeability: Secretory IgA, Lysozyme, and Zonulin

The quantitative determination of fecal markers was performed on stools collected from 41 patients with ASD and 35 CTRLs by ELISA assays. The optical density was determined by a TECAN’s Infinite F50 at 450 nm. Markers’ levels were statistically evaluated using Student’s *t*-test. A *p*-value ≤ 0.05 was considered statistically significant.

#### Secretory IgA

Fecal sIgAs were determined using a RIDASCREEN^®^ sIgA kit according to the manufacturer’s instructions. One hundred mg of stools were weighed and suspended in an extraction buffer, and 100 μL of supernatant processed on the ELISA plate, along with calibrators, negative, low and high positive controls, all of them in duplicate.

#### Zonulin

Zonulin was measured by the Human Zonulin ELISA kit (Bioassay Technology Laboratory, China). Feces were suspended into PBS 1X (DPBS 1X Gibco^®^). Fifty μL of standards, 40 μL of the stool sample, 10 μL of an anti-zonulin antibody, and 50 μL of streptavidin-HRP were added to the plate incubated for 60 min at 37°C.

#### Lysozyme

Fecal lysozyme was assessed using the Lysozyme sandwich ELISA Kit (Immundi-agnostik AG, Cat. No. K 6900) based on two selected antibodies according to the manufacturer’s instructions. Fifteen mg of feces were suspended in 1.5 mL of a sample extraction buffer (IDK extract^®^) and pipetted on a plate.

#### Bacterial DNA Extraction From Stools and 16S rRNA Targeted-Metagenomics

DNA from 41 and 35 fecal samples of ASD and CTRLs subjects, respectively, was extracted using a QIAmp Fast DNA Stool mini kit (Qiagen, Hilden, Germany) according to the manufacturer’s instructions. Amplification of the variable region V3–V4 from the bacterial 16S rRNA gene (∼460 bp) was carried out using the primers 16S_F 5′-(TCG Qaim GCA GCG TCA GAT GTG TAT AAG AGA CAG CCT ACG GGN GGC WGC AG)-3′ and 16S_R 5′-(GTC TCG TGG GCT CGG AGA TGT GTA TAA GAG ACA GGA CTA CHV GGG TAT CTA ATC C)-3′ according to MiSeq rRNA Amplicon Sequencing protocol (Illumina, San Diego, CA, United States). The PCR reaction was set up using the 2 × KAPA Hifi HotStart ready Mix kit (KAPA Biosystems Inc., Wilmington, MA, United States). DNA amplicons were cleaned up by CleanNGS kit beads (CleanNA, Coenecoop 75, PH Wad-dinxveen, Netherlands). A second amplification step was performed to obtain a unique combination of Illumina Nextera XT dual indices for each sample. The final libraries were cleaned up using CleanNGS kit beads, quantified by Quant-iT PicoGreen dsDNA Assay Kit (Thermo Fisher Scientific, Waltham, MA, United States), and normalized to 4 nM. To generate paired-end 250- × −2 bp-length reads, normalized libraries were pooled together and run on the Illumina MiSeq platform according to the manufacturer’s specifications.

### Data Processing and Quality Control

The obtained raw reads were analyzed using Quantitative Insights into Microbial Ecology software (QIIME, 1.9.1) (Caporaso et al., 2010) to perform demultiplexing, quality score checking, low length exclusion, and denoising. Chimeric reads were detected and discarded using USEARCH algorithms (Edgar, 2010). Sequences were grouped into operational taxonomic units (OTUs) by clustering at a threshold of 97% pairwise identity using UCLUST (Edgar, 2010) and submitting representative sequences to PyNAST for sequence alignment (Caporaso et al., 2010). The Greengenes database (v 13.8) was used for OTU matching.

All sequencing data associated with this study were uploaded to the NCBI bioproject database: PRJNA754695.^2^

#### 16S rRNA-Targeted Metagenomics: Statistical Analysis

Unless otherwise stated, all ecological statistical analyses were performed using Python version 3.7. Statistical significance was determined at *p*-value ≤ 0.05 and corrected for multiple hypothesis testing by the false discovery rate (FDR) method Benjamini-Hochberg procedure (Benjamini and Hochberg, 1995). The adjusted *p*-value were reported. The OTU table was normalized by cumulative-sum scaling (CSS) (Paulson et al., 2013). OTUs present in less than.01% of the total sequence count, as well as unassigned taxa, were removed prior to applying statistical analysis. Genus-level comparisons were performed on a reduced matrix of 72 over 461 total OTUs. The criterion for inclusion of variables OTUs reflected their presence in at least 75% of the total sample set.

Alpha diversity statistics was calculated in QIIME (Caporaso et al., 2010) using Shannon, Chao1, Ob-served Species, PD_Whole_Tree, and Goods Coverage diversity indices, and the *p*-value for group comparisons was determined by ANOVA.

Principal coordinate analyses (PCoA) plots were constructed to illustrate the beta diversity of samples based on phylogenetically informed weighted and unweighted Unifrac (Lozupone and Knight, 2005) and Bray–Curtis, Euclidean distance matrices.

To test the association between the covariates and beta diversity measures, permutational analysis of variance (PERMANOVA) was used (Chen et al., 2012) as a distance-based ANOVA method based on permutation (9999 permutations, “Adonis” function in the R “Vegan” package). Linear discriminant analysis effect size (LEfSe) (Segata et al., 2011) was used to determine OTUs characterizing ASDs or CTRLs population. The non-parametric Mann-Whitney U-test and the Wilcoxon signed-rank sum test were used to compare the two independent CTRL and ASD groups. As an initial analysis, some statistics were performed on OTU tables (taxonomic levels 2–6). The interquartile range (IQR), the percentage of zeros (%0), the fold-change (FC), and the *t*-test with relative *p*-value (corrected by the FDR method) were calculated. Applying these filters: IQR ≠ 0,% 0 < 75%, and FC ≠ 0, the variables that were totally unnecessary for classification were discarded.

After applying the filters, further statistics was performed on the remaining variables: for each patient, the OTUs count with abundances > 0 at levels L2–L6, the mean and standard deviation of the abundances, and the Z-test with relative *p*-value between the patients and CTRLs were computed. The Z-score heat maps of OTUs distribution were performed by considering only those with a *p*-value < 0.05. By using Pearson’s correlation coefficient as a metric, clustering of both patients and OTUs was assessed.

To gain more insights into targeted-metagenomics-inferred functions of ASD and CTRLs microbiota, the Phylogenetic Investigation of Communities by Reconstruction of Unobserved States (PICRUSt) v1.1.4 tool was applied (Langille et al., 2013). To get KEGG description, the online database https://www.genome.jp/kegg/ko.html was used (Kanehisa et al., 2016).

In addition, to assess the influence of confounding factors for all comparisons, the Mann-Whitney U-test was applied. Particularly, for the ASD versus CTRLs, comparison confounders, such as gender (male/female) and age (<5 or ≥5 years old), were considered. The confounders taken into account, only for patients, were: age, gender, dietary habits (i.e., no picky eaters/picky eaters), autism severity (low/high-medium symptoms), FS (absence/presence) and GI symptoms (absence/presence). To verify the statistically significant differences between confounders the threshold of the *p*-value for all tests was set at ≤ 0.05.

#### Correlation Analysis on 16S rRNA-Targeted Metagenomics and Clinical Features

To correlate clinical variables and OTUs, Pearson’s correlation coefficient between all variable pairs, with relative *p*-value, was calculated and represented *via* a correlation heat map. Also, for the OTUs, the correlation analysis was performed on all variables and on features passing the filter of *p*-value ≤ 0.05.

#### Machine Learning Models

Multiple machine learning models were trained for the classification task ASD versus CTRLs at each taxonomy level and for the corresponding KOs. The training consisted of a 10-fold cross-validation with a train-test split of 70–30%. To evaluate the model, global and single class accuracies were considered. To extract the feature importance, a permutation performance of 1,000 repetitions was followed. Models tested were: Logistic Regression, SGD Classifier, Logistic Regression CV, Hist Gradient Boosting Classifier, Random Forest Classifier, Extra Trees Classifier, Gradient Boosting Classifier, Bagging Classifier, Ada Boost Classifier, XGB Classifier, XGBRF Classifier, MLP Classifier, Linear SVC, SVC, Gaussian NB, Decision Tree Classifier, Quadratic Discriminant Analysis, K Neighbors Classifier, and Gaussian Process Classifier.

The entire experimental workflow is reported in Figure 1.

## Results

### Subject Characteristics and Clinical Features

Forty-one patients with ASD were vaginally delivered for the 37% (15/41), while the remaining 63% (26/41) were born by cesarean birth. In addition, only 51% (21/41) ASDs were breastfed for over 3 months. The mean values for weight (average, 23 kg ± 10.7), height (average, 116 cm ± 14), and BMI (average, 16.5 kg/m^2^ ± 3.17) were within the normal ranges (Supplementary Table 1, sheet General Characteristics and Figure 1). Indeed, the mean BMI of 16.5 kg/m^2^, assigned to the 75th percentile, classified the ASD children as normal weight (Table 1).

**TABLE 1:** Principal anamnestic characteristics of the patients with ASD and CTRLs.

Anamnestic variables	Children with ASD (*n* = 41)	Neurotypicalchildren (CTRLs) (*n* = 35)	*p*-value
Age year, mean (SD*)	6.5 (3.42)	7.8 (3.62)	0.032
Gender F = female/M = male (SD)	5 (F)/36 (M) (0.33)	14 (F)/21 (M) (0.35)	0.005
BMI (kg/m^2^) for Child and Teen** mean (SD)/percentile	16.50 (3.17)/75th percentile	15.84 (1.85)/50th percentile	0.158
Natural childbirth (frequency%,SD)	15/41 (37%, 0.49)	Nda***	Nda
Breastfeeding > 3 months (frequency%,SD)	21/41 (51%, 0.49)	Nda	Nda

*SD, standard deviation; **Nda: no data associated; ***BMI Percentile Calculator for a Child and a Teen based on CDC growth charts for children and teens aged 2 through 19 years (Kg/m^2^).

The GI symptoms were assessed by using the questionnaire based on Rome IV criteria (Drossman and Hasler, 2016) and specific gastroenterologists’ observations. Based on functional GI disturbances (i.e., constipation, abdominal distension, diarrhea, abdominal pain, GERD, and colic eosinophilia), 73% (30/41) patients with ASD referred the presence of one or more GI symptoms; in detail, 36.6% (15/41) reported only one symptom, 24.4% (10/41) the simultaneous presence of two, 7.3% (3/41) three, and 2.4% four (1/41) and five (1/41) symptoms, respectively. Consequently, 26.8% (11/41) reported the absence of GI symptoms (Supplementary Table 1, Sheet GI symptoms and Figure 1).

Neuropsychological features are reported in Supplementary Table 1 and Figure 1 (Sheet Neuropsychological features). The scores obtained from CBCL Internalizing (CBCL_INT), Externalizing (CBCL_EXT), and Total (CBCL_TOT) Scales evidenced that, in general, a large part of the subjects presented clinical symptoms in CBCL_INT and CBCL_TOT scores, while the large part did not show clinical signs associated with CBCL_EXT scale (Supplementary Table 1, Sheet Neuropsychological features and Figure 1). Particularly, for autism symptoms’ severity, the ASD subjects showed medium for 46.3% (19/41), high for 41.5% (17/41), and low severity for 12.2% (5/41). Regarding CBCL indices (for three patients, the information was missing), those with CBCL_INT reported clinical symptoms for 60.5% (23/38), risk of behavior problems for 18.4% (7/38), and no clinical symptoms for 21.1% (8/38); based on patients with CBCL_EXT, 13.2% (5/38) showed clinical symptoms, 18.4% (7/38) risk of behavior problems, and 68.4% (26/38) no clinical symptoms; lastly, for those with CBCL_TOT, 50% (19/38) indicated clinical symptoms, 23.7% (9/38) risk of behavior problems, and 26.3% (10/38) no clinical symptoms. Remarkably, among the 30 patients with GI symptoms, 28/30 (93%) were also characterized by high (16/30) and medium (12/30) autism symptoms’ severity, respectively, with only 2/30 (7%) with low symptoms.

In addition, the intelligence measures reported as IQ/DQ evidenced that a wide part of the patients with ASD showed cognitive impairment/developmental delay, epilepsy, and drugs exposition (Supplementary Table 1, Sheet Neuropsychological features and Figure 1). In particular, for IQ/DQ measures performed on only 33/41 patients, 36.4% (12/33) were without cognitive impairment/development delay and 63.6% (21/33) reported cognitive impairment/developmental delay.

Finally, 85.4% (35/41) and 92.7% (38/41) of the patients with ASD referred to no drugs’ consumption or epilepsy, respectively (Supplementary Table 1, Sheet Neuropsychological features and Figure 1). The remaining 6/41 patients were administrated with drugs, such as Topiramate, Depakin, Abilify (antipsychotic drug, 3rd generation), Risperdal (antipsychotic drug, 2nd generation) (Supplementary Table 1, Sheet Neuropsychological features and Figure 1). Moreover, collected information on dietary needs revealed that 24% (10/41) of ASD referred to FS, 24% (10/41) followed by gluten- and 32% (13/41) casein-free diet. 17% (7/41) were exposed to probiotics’ and 24% (10/41) to antibiotics’ administration (Supplementary Table 1, Sheet Nutritional habits and probiotics/antibiotic administration and Figure 1).

### Inflammation and Intestinal Permeability Markers

**FIGURE 2:**
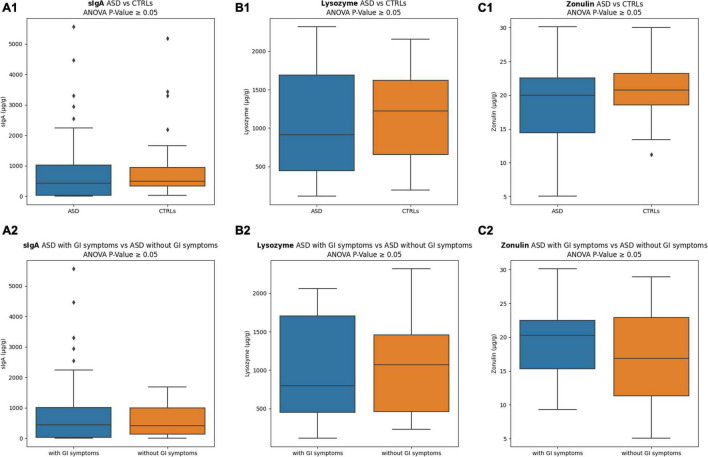
Inflammation and intestinal permeability markers. **(A1)** sIgA comparison between ASD versus CTRLs; **(A2)** sIgA comparison between ASD with GI symptoms versus ASD without GI symptoms; **(B1)** lysozyme comparison between ASD versus CTRLs; **(B2)** lysozyme comparison between ASD with GI symptoms versus ASD without GI symptoms; **(C1)** zonulin comparison between ASD versus CTRLs; **(C2)** zonulin comparison between ASD with GI symptoms versus ASD without GI symptoms. The interquartile range is represented by the box, and the line in the box is the median. The whiskers highest and lowest data points are reported, while the dots represent the outliers.

Levels of sIgA, lysozyme, and zonulin (μg/g), measured in stools of ASD and CTRLs subjects, are reported in Figure 2. The sIgA levels were comparable in the two groups; the lysozyme was higher for the CTRLs subjects, while zonulin appeared lower for the patients with ASD. No statistically significant differences were observed between ASD versus CTRLs (*p*-value ≥ 0.05). However, stratifying the patients with ASD into two subgroups based on GI symptoms presence/absence, remarkable differences were identified for both lysozyme and zonulin markers, showing in the ASD with GI symptoms a decreased median value for lysozyme (799.86 ± 672.91) (Figure 2B2) and an increased median value for zonulin (20.28 ± 5.21) (Figure 2C2), regardless of absence of statistically significant (*p*-value ≥ 0.05). Conversely, the sIgA levels were comparable in the two ASD subgroups (Figures 2A1–C1,A2–C2). Moreover, to exclude the influence of other factors, the comparisons of ASD with GI symptoms versus CTRLs and ASD with high/moderate autism symptoms versus CTRLs were performed, but any comparison resulted in statistically significant (*p*-value ≥ 0.05) (Supplementary Figure 2).

### Gut Microbiota Ecology

After data filtering, a total of 9,525,477 sequence reads of 16S rRNA gene amplicons were obtained with an average of 70,040 reads/sample and an average length of 487 bp (calculated after primer removal). Resulting OTUs were detected by sequence-database matching (Supplementary Table 2).

The GM composition of ASD and CTRLs groups was analyzed by ecological analysis as α- and β-diversity. Statistically significant differences in the α-diversity were found in all the comparisons, except for Good Coverage and Shannon index, showing lower values of diversity for patients with ASD (Supplementary Figure 3).

**FIGURE 3:**
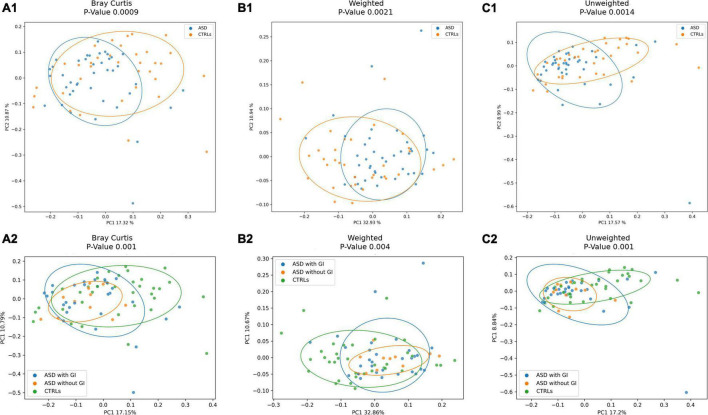
Beta-diversity ecological analysis. Principal Coordinate Analysis (PCoA) plots shows Bray Curtis in **(A)**. **(A1)**, patients with ASD versus CTRLs; **(A2)**, patients with ASD grouped for presence of GI (with GI) or GI absence (without GI) symptoms versus CTRLs cohorts. Weighted UniFrac algorithms in **(B)**. **(B1)**, patients with ASD versus CTRLs; **(B2)**, patients with ASD grouped for presence of GI (with GI) or GI absence (without GI) symptoms versus CTRLs cohorts. Unweighted UniFrac algorithms in **(C)**. **(C1)**, patients with ASD versus CTRLs; **(C2)**, patients with ASD grouped for presence of GI (with GI) or GI absence (without GI) symptoms versus CTRLs cohorts.

The ecological analysis assessed by β-diversity algorithms provided statistically significant (*p*-value ≤ 0.05) differences between patients with ASD and CTRLs (Figure 3).

**FIGURE 4:**
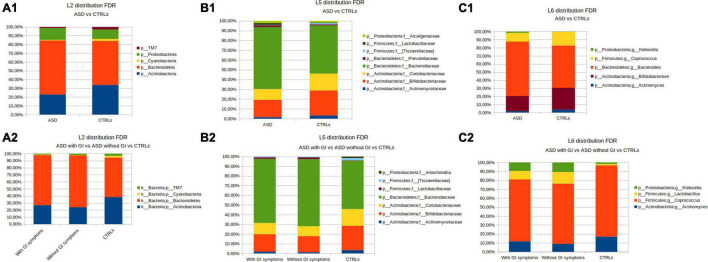
OTUs distribution performed by the Mann–Whitney U-test. **(A)** The phylum level; **(A1)** the patients with ASD versus CTRLs; **(A2)** the patients with ASD grouped for presence of GI (with GI) or GI absence (without GI) symptoms versus CTRLs cohorts. **(B)** The family level; **(B1)** the patients with ASD versus CTRLs; **(B2)** the patients with ASD grouped for presence of GI (with GI) or GI absence (without GI) symptoms versus CTRLs cohorts. **(C)** The genus level; **(C1)** ASD versus CTRLs; **(C2)** the patients with ASD grouped for presence of GI (with GI) or GI absence (without GI) symptoms versus CTRLs. Only statistically significant OTUs (Mann-Whitney U *p*-value FDR ≤ 0.05) were represented.

At the phylum level (L2), for the coupled ASD versus CTRLs comparison, the OTUs abundance differences relied on Actinobacteria, Cyanobacteria, and TM7, which were higher in the CTRLs group, and on Proteobacteria and Bacteroidetes increase in ASD (Figure 4A1). By grouping the patients according to presence/absence of GI symptoms, a higher abundance of Bacteroidetes was observed in the ASD without GI symptoms group (Figure 4A2).

At the family level (L5), Alcaligenaceae, Lactobacillaceae, Prevotellaceaeae, and Bacteroidaceae resulted in more abundant in ASD, while, in the CTRLs subjects, Coriobacteriaceae, Bifidobacteriaceae, Actynomicetaceae, and (Tissierellaceae) were higher than in ASDs (Figure 4B1). Bacteroidaceae and Lactobacillaceae were higher in ASD without GI symptoms, while Coriobacteriaceae, Bifidobacteriaceae, (Tissierellaceae), and Actynomicetaceae appeared to increase in CTRLs (Figure 4B2).

At the genus level (L6), *Bacteroides* and *Klebsiella* were more abundant in ASDs than in CTRLs, while *Coprococcus, Bifidobacterium*, and *Actinomyces* were higher in CTRLs (Figure 4C1). In particular, the detail of the five statistically significant OTUs comparisons for ASD versus CTRL at the genus level is reported in Supplementary Figure 4. In the separation of the patients with ASD by presence/absence of GI symptoms, the comparison with CTRLs highlighted that *Klebsiella* and *Lactobacillus* were higher in the ASD without GI symptoms (Figure 4C2).

Ecological analysis in terms of α- and β-diversity and global OTUs distribution was also performed for the patients with ASD grouped according to (i) autism symptoms severity, (high/moderate and low) (Supplementary Figures 5–7); (ii) FS (presence/absence) (Supplementary Figures 8–10); and (iii) picky eaters (PEs) (presence of FS and/or gluten/casein-free diet) and no-PE (absence of FS and/or gluten/casein-free diet) (Supplementary Figures 11–13) in order to evaluate all variables affecting GM composition.

By considering these clinical and anamnestic features, both α-diversity and β-diversity were notable to discriminate any of the ASD-stratified subgroups.

In addition, the influence of confounding factors was considered. Firstly, in the comparison of ASD versus CTRLs, age and gender statistically affected the differences between the two groups (Supplementary Table 3). Thus, to investigate the effect of age, the subjects were stratified into two groups [<0–5 years old (23 ASD; 11 CTRLs) and ≥5 years old (18 ASD; 24 CTRLs)]. Indeed, the α- and β-diversity and the global OTUs distribution showed statistically significant differences between the patients and CTRLs (Supplementary Figures 14–16). Particularly, α-diversity showed differences (*p*-value ≤ 0.05) between ASD ≤ 5 versus CTRLs ≤ 5 years old and, also, versus the entire group of CRTLs (Supplementary Figures 14A1–D1,A2–D2).

Also, based on age subgroups’ comparison, the OTUs distribution showed for Bacteroidetes and Actinobacteria statistically significant differences (*p*-value ≤ 0.05) (Supplementary Figure 16A). Moreover, by considering the comparison obtained by the Mann-Whitney U-test on the entire group of CTRLs versus ASD grouped by age, Cyanobacteria and TM7 were statistically different for ASD < 5 versus CTRLs (Supplementary Figure 16B), while Cyanobacteria, Proteobacteria, Bacteroidetes, and Actinobacteria resulted in statistically different (*p*-value ≤ 0.05) for the comparison between ASD ≥ 5 versus CTRLs (Supplementary Figure 16B). At the family level, Gemellaceae, Streptococcaceae, Erysipelothricaceae, Carnobacteriaceae, Bifidobacteriaceae, and Actinomycetaceae were statistically different for ASD < 5 versus CTRL (Supplementary Figure 16C). In addition, for the comparison referred to the entire subgroups’ set obtained by the Mann-Whitney U-test, Alcaligenaceae, Prevotellaceae, Bacteroidaceae, Ciriobacteriaceae, Actinomycetaceae, Erysipelotrichaceae, and Bifidobacteriaceae resulted in statistically different (*p*-value ≤ 0.05) only for the comparison between ASD ≥ 5 versus CTRLs (Supplementary Figure 16D).

Moreover, by considering the ASD subgroups characterized in terms of dietary habits and FS, confounders’ analysis highlighted statistical significance in their comparison (Supplementary Table 3). However, adjusted results did not provide for further GM comparisons or any additional information for low sample sizing (data not shown).

#### Microbial Biomarkers Predictive Analysis: Linear Discriminant Analysis Effect Size

**FIGURE 5:**
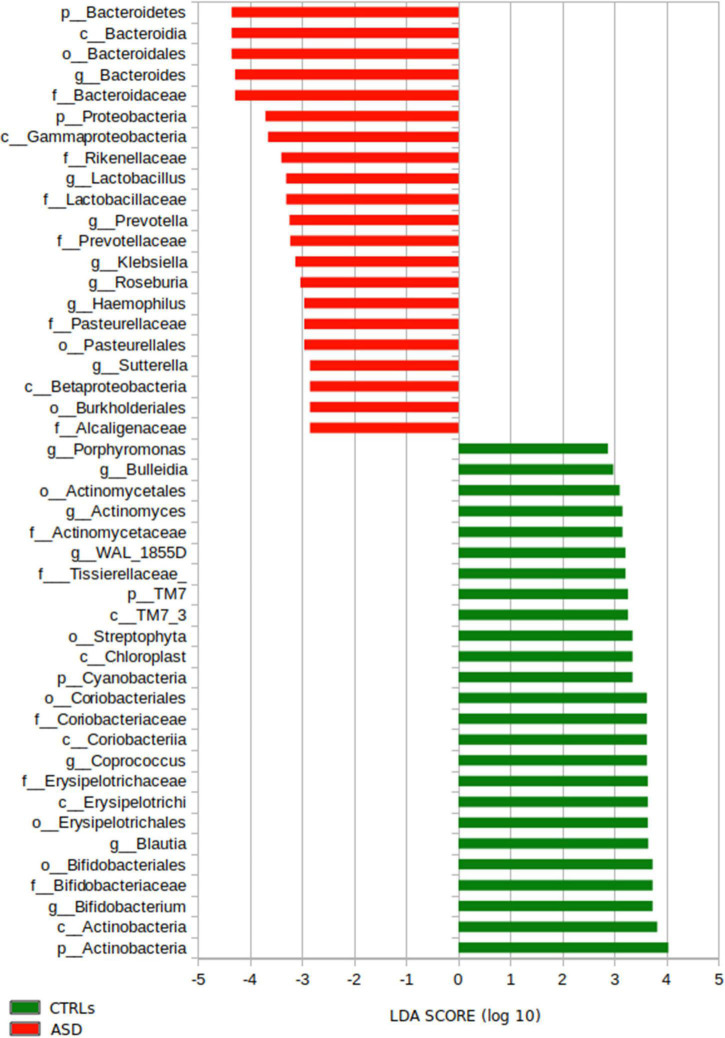
LEfSe analysis for the comparison between the patients with ASD and CTRLs cohorts’ gut microbiota. Histogram represents the LDA scores until L6 taxonomy filtered by statistical significance between the two groups. In red and in green are represented microbial biomarkers for the patients with ASD and the CTRLs subjects, respectively.

To infer taxonomic differences between the GM of the patients with ASD and CTRLs in terms of potential biomarkers, a linear discriminant analysis (LDA) effect size (LEfSe) algorithm (cutoff ≥ 2) was performed and 49 top-ranking OTUs characterizing the microbiota were identified (Figure 5).

In particular, the analysis provided ASD-related microbial biomarkers (*p*-value ≤ 0.05), such as Bacteroidetes, Proteobacteria (L2); Bacteroidaceae, Rikenellaceae Lactobacillaceae, Prevotellaceae, Pasteurellaceae, and Alcaligenaceae (L5); *Bacteroides*, *Lactobacillus, Prevotella, Klebsiella, Roseburia, Haemophilus*, and *Sutterella* (L6) (Figure 5).

On the other hand, the GM of CTRLs was characterized (*p*-value < 0.05) by TM7, Cyanobacteria, and Actinobacteria (L2); Tissierellaceae, Coriobacteriaceae, Erysipelotrichaceae, and Bifidobacteriaceae (L5); *Porphyromonas, Bulleidia, Actinomyces, WAL_1855D, Coprococcus, Blautia*, and *Bifidobacterium* (L6) (Figure 5).

**FIGURE 6:**
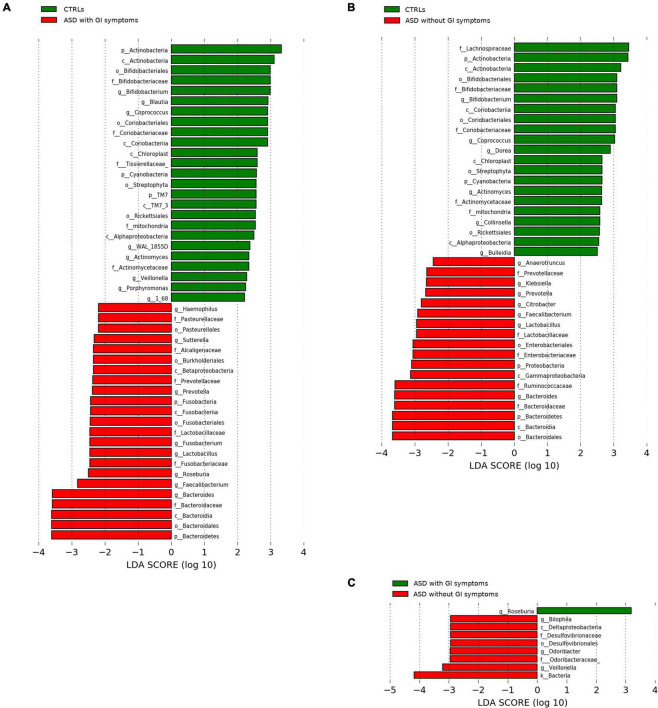
LEfSe analysis between the patients with ASD grouped for presence of GI (with GI) or GI absence (without GI) and CTRLs cohorts. Histogram represents the LDA scores of bacteria (L6) with statistically significant differential abundance between groups. **(A)** CTRLs versus ASD with GI symptoms; **(B)** CTRLs versus ASD without GI symptoms; **(C)** ASD with GI versus ASD without GI symptoms. In red, ASD; green, CTRLs. All OTUs reported were statistically significant (*p*-value ≤ 0.05).

Moreover, the microbial predictive analysis was applied to ASDs grouped for presence/absence of GI symptoms versus CTRLs (Figures 6A,B). Significant differences in the comparison between ASD with GI symptoms versus CTRLs relied on the ASD biomarkers *Haemophilus*, Pasteurellaceae, *Roseburia*, *Fusobacterium*, and Enterobacteriaceae (Figure 6A), while, for the ASD subgroup without GI symptoms compared to CTRLs, Proteobacteria, Gammaproteobacteria, Ruminococcaceae, and *Klebsiella* (Figure 6B) were associated with ASD. On the contrary, for CTRLs, the highest scored microbial signatures were *Blautia* and *Veillonella* in the comparison versus ASD with GI symptoms, while *Colinsella, Bulleidia*, and *Dorea* better-differentiated CTRLs in the comparison versus ASD without GI symptoms (Figures 6A,B). Regarding the comparison between ASD with GI versus ASD without GI symptoms, *Bilophila*, *Odoribacter*, and *Veillonella* characterized ASD without GI, while ASD with GI symptoms was characterized by *Roseburia* (Figure 6C).

Moreover, Gram-positive bacteria, such as Ruminococcaceae, resulted higher in the LEfSe analysis of the patients with ASD in the absence of GI symptoms when compared to CTRLs, while the abundance of Ruminococcaceae in PE/noPE and FS/noFS was comparable (data not shown).

#### Predictive Functional Analysis

**FIGURE 7:**
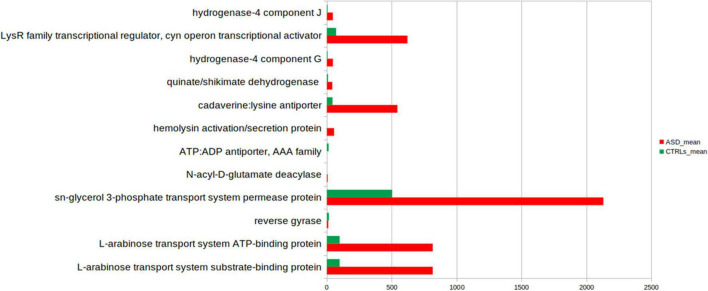
Kyoto Encyclopedia of Genes and Genomes (KEGG) biomarkers inferred from the whole set of OTUs of the patients with ASD and the CTLRs subjects. A linear discriminant effect size (LEfSe) analysis was performed [α = 0.05, a logarithmic discriminant analysis (LDA)] score threshold = 2.0). ASD- and CTRLs-related KEGGs entries are represented in red and green bars, respectively.

Phylogenetic Investigation of Communities by Reconstruction of Unobserved States based on reference OTUs was applied to predict abundances of the KEGG orthologs (KOs) (Figure 7).

Few KOs showed significant differences in the GM of ASD and the CTRLs subjects (*p*-value ≤ 0.05). Twelve metabolic microbial pathways differentially expressed were related to 46 selected OTUs, and 10/12 were linked to patients with ASD (Figure 7). In particular, the top 10 pathways were hydrogenase-4 component J; LysR family transcriptional regulator cyn operon transcriptional activator; hydrogenase-4 component G; quinate/shikimate dehydrogenase; cadaverine:lysine antiporter; hemolysin activation/secretion protein; N-acyl-D-glutamate deacylase; sn-glycerol 3-phosphate transport system permease protein; L-arabinose transport system ATP-binding protein; L-arabinose transport system substrate-binding protein (Figure 7 and Table 2).

**TABLE 2:** Overexpressed KEEG pathways in the patients with ASD and CTRLs.

Class	Subclass	KEGG pathways	Overexpression
Brite Hierarchies	Transcription factors	LysR family transcriptional regulator, cyn operon transcriptional activator	ASD
Metabolism	Enzymes with EC numbers	Hydrogenase-4 component G	ASD
Metabolism	Phenylalanine, tyrosine, and tryptophan biosynthesis	Quinate/shikimate dehydrogenase	ASD
Brite Hierarchies	Transporters	Cadaverine:lysineantiporter	ASD
Environmental Information Processing	Bacterial secretion system	Hemolysin activation/secretion protein	ASD
Metabolism	Enzymes with EC numbers	*N*-acyl-d-glutamate deacylase	ASD
Environmental Information Processing	ABC transporters	Sn-glycerol 3-phosphate transport system permease protein	ASD
Environmental Information Processing	ABC transporters	l-arabinose transport system ATP-binding protein	ASD
Environmental Information Processing	ABC transporters	l-arabinose transport system substrate-binding protein	ASD
DNA replication proteins	DNA Ttopoisomerases	Reverse girase	CTRL

**FIGURE 8:**
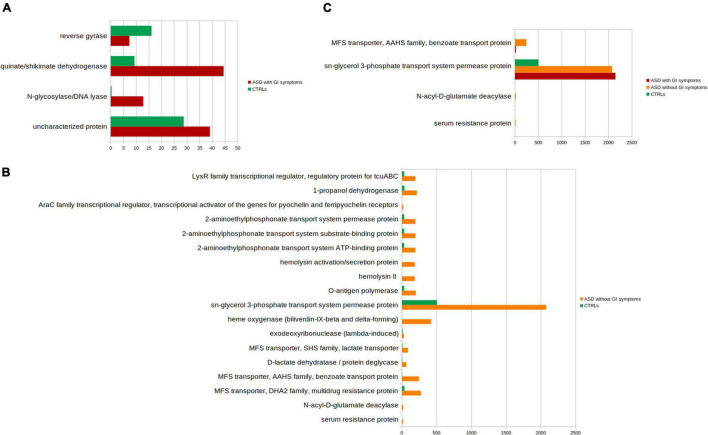
Kyoto Encyclopedia of Genes and Genomes (KEGG) biomarkers inferred from the whole set of OTUs of the patients with ASD grouped into the patients with GI and without GI symptoms versus the CTRLs subjects. KOs distribution for each group computed by the Kruskal-Wallis test (*p*-value < 0.001 and relative abundances > 10). **(A)** ASD with GI symptoms versus CTRLs; **(B)** ASD without GI symptoms CTRLs; **(C)** ASD with GI versus ASD without GI symptoms and versus CTRLs. In red, ASD with GI symptoms; orange, ASD without GI symptoms; green, CTRLs.

The PICRUSt analysis was also applied to ASDs subgroups compared to CTRLs (Figures 8A–C and Supplementary Tables 4–6). Significant differences (*p*-value ≤ 0.05) in the comparison between ASD with GI symptoms versus CTRLs particularly relied on quinate/shikimate dehydrogenase and N-glycosylase/DNA lyase for ASDs and reverse gyrase for CTRLs (Figure 8A and Supplementary Table 4).

The comparison ASD without GI symptoms versus CTRLs allowed us to predict 14 overexpressed ASD-related metabolic pathways (Figure 8B and Supplementary Table 5), including sn-glycerol 3-phosphate transport system permease protein; cadaverine: lysine antiporter; 1-propanol dehydrogenase; and N-acyl-D-glutamate deacylase.

Lastly, the comparison of ASD with GI symptoms versus ASD without GI symptoms and versus CTRLs identified the sn-glycerol 3-phosphate transport system permease protein associated with both ASD with/without GI symptoms (Figure 8C and Supplementary Table 6).

The comparison between ASD with GI symptoms versus ASD without GI symptoms did not show statistically significant differences.

#### Hierarchical Clustering of Subjects According to Operational Taxonomic Unit Distribution

Global OTUs distribution differences between ASD and CTRLs were evaluated by hierarchical clustering in Supplementary Figure 17. The ASD and CTRL groups displayed different microbial profiles at L2 (Supplementary Figure 17A), revealing two main clusters (namely, A and B). The major part of the patients with ASD belonged to the Cluster B and was characterized by the highest abundance of Bacteroidetes and Proteobacteria. Conversely, the Cluster A was predominantly constituted by the CTRL group and represented by a major abundance of Firmicutes, Cyanobacteria, Actinobacteria, and TM7.

At L6, the heatmap showed clear separation between ASD and CTRL, displaying different microbial profiles (Supplementary Figure 17B). The Cluster A, mainly composed of the patients with ASD, was characterized by an increase in *Bacteroides*, *Sutterella*, *Lactobacillus*, *Prevotella*, *Staphylococcus*, and *Haemophilus*. On the contrary, the CTRLs mainly included in Cluster B showed an increased level of WAL_1855d, Coriobacteriaceae, *Bifidobacterium*, *Streptophyta*, Lachnospiraceae, *Coprococcus*, TM7-3, Gemellaceae, *Eggerthella*, Eryspelotrichaceae, *Blautia*, and *Ruminococcus* belonging to the Lachnosperaceae family, *Actinomyces*, and *Streptococcus*.

#### Correlation Network Analysis of Autism Spectrum Disorder Clinical Features and Gut Microbiota Ecology

**FIGURE 9:**
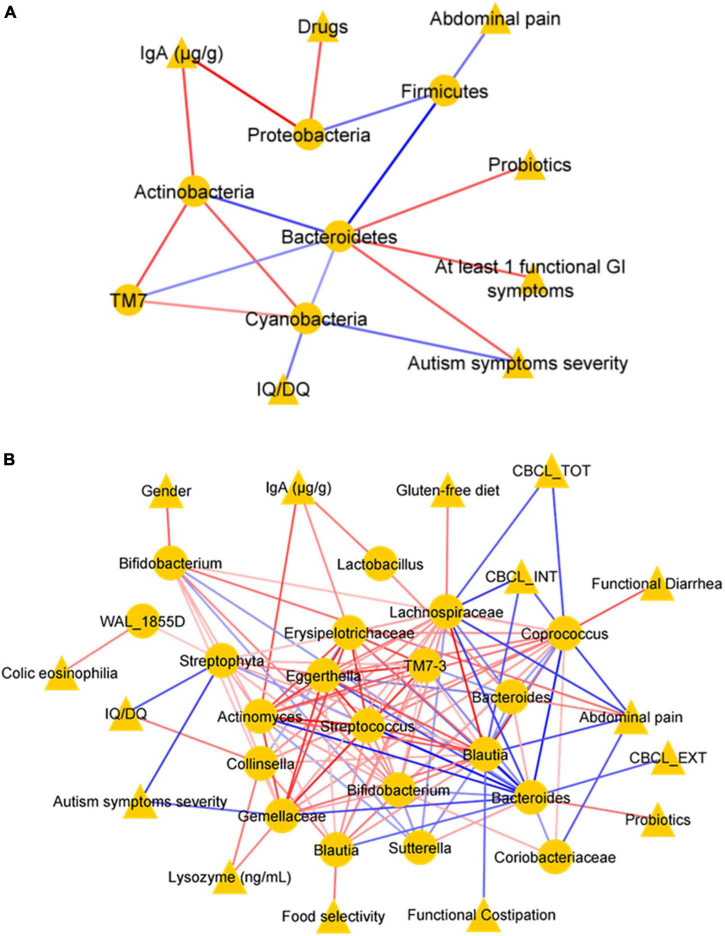
Correlation networks. Correlation analysis between OTUs (selected with the *t*-test with *p*-value ≤ 0.05) and clinical variables at the phylum level (L2) **(A)** and at the genus level (L6) **(B)**. In each network, nodes represent the OTUs (circles) and the clinical variables (triangles), and an edge between two nodes occurs if they exhibit a statistically significant correlation (*p*-value ≤ 0.05). The color of the network edges indicates positive (red) and negative (blue) correlations.

To explore the possible relationship between clinical features and GM composition in ASD children, a correlation network was generated by Pearson’s correlation coefficient (Figure 9). At the phylum level (L2) (Figure 9A), the correlation analysis showed a significant statistically positive correlation (*p*-value ≤ 0.05) between few clinical variables and OTUs. Particularly, sIgA positively correlated with Proteobacteria and Actinobacteria, and drugs administration with Proteobacteria. In addition, at least one functional GI symptom, probiotics, and autism symptoms’ severity correlated positively with Bacteroidetes. On the contrary, the analysis showed a negative correlation between abdominal pain and Firmicutes; autism symptoms severity, IQ/DQ, and Cyanobacteria (Figure 9A).

In addition, at L6, the analysis showed a statistically significant (*p*-value ≤ 0.05) positive correlation between paired clinical variables and OTUs, such as lysozyme concentration and Gemellaceae; sIgA and Erysipelotrichaceae, antibiotic therapy, and *Prevotella*; diarrhea and *Coprococcus*; gender and *Prevotella*; casein-free diet and S*taphylococcus*; colic eosinophilia and WAL_1855D; gluten-free diet and Lachnospiraceae; abdominal functional distention and *Prevotella*; probiotics and *Bacteroides*; and between OTUs-OTUs, such as *Lactobacillus* and Actinomyces. Conversely, abdominal pain showed negative correlations with Coriobacteriaceae, *Coprococcus*, *Blautia*, and Lachnospiraceae; constipation with *Staphylococcus* (Figure 9B). In addition, almost all neuropsychological features showed statistically significant negative correlations (*p*-value < 0.05): autism symptoms’ severity with Gemellaceae and Streptophyta; CBCL_TOT and CBCL_EXT with Lachnospiraceae and *Bacteroides*; CBCL_INT with *Ruminococcus*, Lachnospiraceae, except for a positive correlation with *Prevotella;* autism symptoms severity and IQ/DQ with Streptophyta (Figure 9B).

### Model Classifications Analysis Based on Operational Taxonomic Units and KEGG Orthologs Pathways’ Features

**FIGURE 10:**
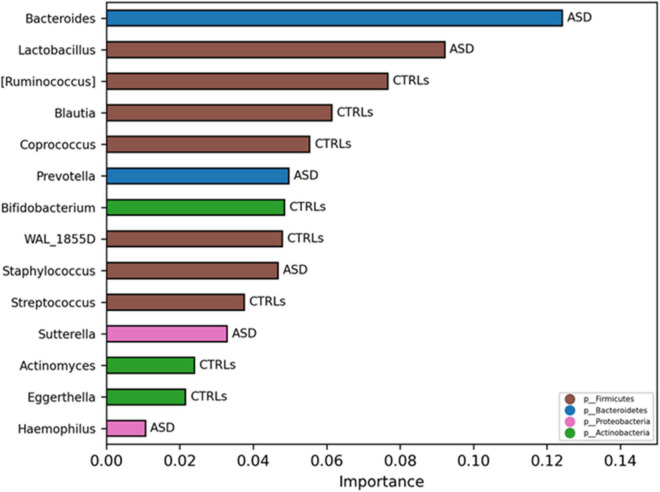
Significant OTUs selected by classification model analysis. The bars represent the importance scores of each OTU in the ASD prediction models versus CTRLs.

To investigate if the GM of the patients with ASD could be predictive of a disease phenotype, a model classification analysis based on machine learning (ML) was used. By this analysis, the most important features of the GM were selected at L6, revealing that the GM ecology was able to classify the 80% of the patients with ASD compared to CTRLs based on the two models: Quadratic Discriminant Analysis and Gaussian Process Classifier (Supplementary Table 7). The specific selected OTUs for ASD and CTRLs obtained by the consistent models are reported in Figure 10. Contextually, a model classification analysis based on ML identified both KOs and ko pathways able to classify the 73% of the patients with ASD versus CTRLs (*p*-value ≤ 0.05). The flagellar assembly (ko02040) resulted in the best KO pathway, describing ASDs (Supplementary Figure 18).

## Discussion

Several aspects of individuals’ behavior and physiology can be affected by changes in the host genome and microbiome (Sharon et al., 2016; Vuong and Hsiao, 2017; Lynch and Hsiao, 2019; Sherwin et al., 2019), and their interactions (Fischbach and Segre, 2016); hence, it is crucial to understand whether ASD symptoms are influenced by modifications of the host’s GM-brain axis. Indeed, growing pieces of evidence indicate that environmental factors, including diet, family habits, etc., may play an important role in the ASD (Roussin et al., 2020; David et al., 2021; Yap et al., 2021).

Particularly, GI functional symptoms, such as constipation, abdominal pain, flatulence, and diarrhea, have been described as representing an important comorbidity (Liu S. et al., 2019) in the wide autism phenotype, if compared with children with typical development (Chaidez et al., 2014). Hence, gut inflammation related to intestinal barrier integrity and innate immune barrier defense exerted as antimicrobial action against Gram-positive bacteria were herein evaluated, determining fecal sIgAs, zonulin, and lysozyme titers.

In our dataset, the median levels of sIgA were slightly higher in stool samples of CTRLs compared with ASD, suggesting lower levels of IgA in these patients, possibly associated with impaired gut immunity (Zhou et al., 2018). Interestingly, low substantial inflammation may be due to a specific turn-over of *Sutterella* species (Kaakoush, 2020), identified as microbial biomarkers of the patients with ASD by LEfSe analysis and corroborated by comparable values of IgA for both the patients with ASD in presence/absence of GI symptoms.

Also median value of lysozyme appeared higher in the CTRLs group compared to ASD, with a further decrease in the patients with GI symptoms, as previously observed (Adams et al., 2011). Worth noting is that the statistically significant presence of *Ruminococcus* in ASD without GI symptoms may corroborate the idea that a starch-degrading bacterium (Lordan et al., 2020) could improve the GI wellness, preserving from symptoms, more responding to nutritional triggers rather than to host innate immunity (Yap et al., 2021). Typical ASD profiles, associated with GI symptoms and behavior indices, may be correlated with ASD severity (Holingue et al., 2018), hence suggesting that the communication between gut-brain axis and nutritional altered habits may be involved in ASD pathogenesis (Mayer et al., 2015; Beaudet, 2017). Moreover, the presence of *Roseburia* as the microbial biomarker of the patients with ASD, also in the presence/absence of GI symptoms, compared to CTRLs, may, indeed, contrast the idea that SCFAs-producing bacteria may preserve the microbiome ecology and the defense against pathogenic bacteria, without the innate immunity response of lysozyme (Adams et al., 2011).

The zonulin is involved in the intestinal permeability regulation and has been reported as reducing the expression of barrier components in the tight junctions in 75% of the patients with ASD (Fiorentino et al., 2016; Özyurt et al., 2018). In addition, zonulin is a precursor of haptoglobin 2 (Hp2) (Tripathi et al., 2009), and some previous works have suggested that the presence of the haptoglobin 2 allele could be related to a higher risk of immune-mediated diseases development (Blum et al., 2008).

However, Rose et al. (2018) observed no changes in zonulin levels in ASDs with no statistically significant over-expression of the HP2 gene product in the patients with ASD, but only a significant difference between ASD with GI symptoms and typically developing children with GI symptoms.

However, in this latter case, it is remarkable to underline that the zonulin comparison between ASDs and CTRLs subjects was meaningless based on the lack of linearity between intestinal permeability and fecal zonulin concentration in the normal-weight CTRLs subjects (Seethaler et al., 2021).

The high/medium autism severity and the presence of at least one of the GI symptoms in our patients resulted intrinsically related, possibly suggesting the GI symptom phenotype as the driving force of the underlying neuropsychological phenotype.

About global microbial distribution, the Pasteurellaceae family (Liu F. et al., 2019) was significantly associated with patients with ASD reinforcing the idea of a link to host inflammation (Shin et al., 2015). Indeed, the outgrowth of Proteobacteria promotes intestinal inflammation driven by dysregulated innate immune responses, as demonstrated by studies in mice models with genotypes influencing the adaptive immunity (Shin et al., 2015).

Moreover, the data suggested that the observed GM ecological profiles were specific for the subject affected by ASD, dependently of age, in agreement to what happens in children affected by neuropsychiatric syndromes related to pediatric autoimmune neuropsychiatric disorders associated with streptococcal infections syndrome (PANDAS), in which the GM ecological analyses showed the presence of two clear subject clusters based on the age range (Quagliariello et al., 2018).

In addition, Bacteroidetes were predominantly associated with the patients with GI symptoms underlying autism symptoms severity, as previously reported (Morais et al., 2021). Indeed, a cross-sectional study (Christian et al., 2015) reported important gut microbial differences in terms of OTUs diversity and abundance related to maternal ratings of toddlers temperament, especially among boys. In particular, the major discriminant variable was, indeed, represented by the Bacteroidetes Phylum that could be associated with infant temperament.

Our patient’s cohort was composed prevalently by males, which represent one of the variables that affect their gut microbial profile, and there are also pieces of strong evidence (Tamana et al., 2021) of positive relationships between Bacteroidetes in late infancy and subsequent neurodevelopment, most prominently among males. Particularly, concentrations of propionic acid produced by *Bacteroides* may play beneficial effects for the enterocytes despite of high concentrations may be related to toxic effects (Frye et al., 2016; Srikantha and Mohajeri, 2019). Moreover, *Bacteroides fragilis* showed a pathological role described in neurodevelopment processes, including ASDs (Nagaraju et al., 2019).

Besides *Bacteroides* (De Angelis et al., 2013; MacFabe, 2015), also, *Roseburia* was detected in higher abundance in the patients with ASD compared to CTRLs; interestingly, *Roseburia* degrades starch and ferments other carbohydrates to synthesize SCFAs.

Particularly, propionic acids and other SCFAs have different impacts on cellular and mitochondrial activity, which could include also the modulation of T-cell function and cytokine production (Rose et al., 2017) in a concentration-dependent manner (Frye et al., 2015). Hence, SCFAs could disrupt cellular physiology to cause functional GI disease related to ASD, such as dismotility and non-specific inflammation (Kang et al., 2017; Rose et al., 2017).

Moreover, the role of *Roseburia* of being a microbial biomarker of patients with ASD with GI symptoms, characterized by lysozyme defect, compared to the patients without GI symptoms, may, indeed, question the idea that SCFAs-producing bacteria may preserve the GM ecology (Tamanai-Shacoori et al., 2017) without the innate immunity response of lysozyme. On the other hand, the statistically significant presence of *Ruminococcus* (Ruminococcaceae) in ASD without GI symptoms may corroborate the idea that a starch-degrading bacterium may, in any case, improve the GI wellness, preserving from symptoms, and responding to nutritional triggers rather than to host innate immunity.

Moreover, *Roseburia* has a low capacity to degrade free amino acids (FAAs) at the gut level and, because, in ASD, the especially increased FAA has been described to be glutamate, probably its increase is related to high concentration of *Roseburia* (De Angelis et al., 2013). Indeed, glutamate acts as neurotransmitters in the CNS, and, since it is a potent neurotoxin, an excess could lead to neuronal death, as reported in neuropsychiatric disorders pathophysiology, including autism (Won et al., 2012; Yang and Chang, 2014). Consistently, De Angelis et al. (2015) determined FAAs in fecal samples of the patients with ASD associated with the presence of proteolytic bacteria, such as *Bacteroides* and *Roseburia.*

*Prevotella* and *Lactobacillus* were also higher in all the patients with ASD, compared to CTRLs. Previous research has indicated a correlation between dietary habits and high levels of *Prevotella* spp. in patients with ASD. High abundance of *Prevotella* spp. affects inflammation and mucosal and systemic T cell stimulation (Larsen, 2017; Iljazovic et al., 2021). However, *Prevotella* is also involved in the polysaccharides degradation and fermentation, leading to the production of SCFAs (Williams et al., 2017; Gálvez et al., 2020).

*Sutterella* distribution was higher in the GM of ASDs with GI symptoms, corroborating previous studies (Williams et al., 2012). ML actually identified this microbe as ASD features in the disease classification model.

On the contrary, beneficial bacteria, such as *Bifidobacterium*, decreased in all the ASD subjects regardless of presence or absence of GI symptoms, compared to CTRLs, thus reducing its protection activity against gut inflammation and cooperating in the development of the immune system (Medina et al., 2008; Salazar et al., 2008; Hashemi et al., 2017). The decrease of Bifidobacteria in the GM of ASD children may trigger rearranging of important microbes, potentially contributing to some ASD symptoms (Averina et al., 2020). Recently, *Bifidobacterium* has been proposed as a psycobiotics due to its ability to generate neuromodulators and to affect gut–brain relations by the interaction with other commensal microbes (Sarkar et al., 2016).

Consistently, with our results, Kang et al. (2013) detected a significantly higher abundance of *Coprococcus* in CTRLs compared to children with ASD. The reduction of *Veillonella* and *Blautia* in the children with ASD, regardless of GI symptoms, may be potentially linked to the presence of ASD severity symptoms rather than to GI symptoms severity or specific diet/supplement habits (i.e., FS, gluten- and casein-free diet). Thus, the reduction in ASD of these beneficial microbes might be involved in the pathogenesis of disease (Liu S. et al., 2019), indirectly influencing social brain functions (Kang et al., 2013). Conversely, the exceeding amount of ugly bacteria may increase the presence of toxic microbial by-products, passing through an irregular porous blood–brain barrier and entering into the bloodstream and the brain (Fiorentino et al., 2016).

One of the predicted biochemical pathways overexpressed in the children with ASD was the quinate/shikimate dehydrogenase, known to catabolize quinate and shikimate in Gram-negative bacteria, such as Proteobacteria, to produce aromatic AA, such as L-phenylalanine, L-tyrosine, and L-tryptophan, which represents molecular building blocks for protein biosynthesis (Herrmann and Weaver, 1999).

In addition, the sn-glycerol 3-phosphate transport system permease protein represented an overexpressed pathway in ASD. It is involved in the incorporation of exogenous FAs into phosphatidylethanolamine in Gram-negative bacteria belonging to Proteobacteria phylum (Rock and Jackowski, 1985).

The other highly represented biochemical pathways were the cadaverine lysine antiporter that represents a signaling system to acid stress in different γ-Proteobacteria (Brameyer et al., 2020); the bacterial 1-propanol dehydrogenase, associated with alcohol dehydrogenase (ADH), is able to attenuate alcohol toxicity and modulate alcohols’ interaction at the GM level (Ferraro and Finkel, 2019; Lin et al., 2021); the N-Acyl-D-glutamate amidohydrolase is produced by Gram-negative bacteria and derived from D isomers of FAAs, such as N-acetylglutamate, glutamate, aspartate, and asparagine (Sakai et al., 1991).

As diet represents a major reservoir of circulating metabolites, its modulation has been proposed to modulate intricate behaviors (Needham et al., 2018a,b, 2021; Pulikkan et al., 2019), including ASDs (David et al., 2021; Yap et al., 2021).

Moreover, the research is moving toward the understanding of GM-related metaproteomics (Levi Mortera et al., 2022) and metabolomics and microbiota-host crosstalk, trying to describe the functional ASD-related enterophenotype. In this context, the nutritional role needs to be further investigated by novel approaches, such as foodomics (Putignani and Dallapiccola, 2016), applied to patient cohorts from different geographical settings and social environments. In addition, the role of host-related immunological biomarkers and the gut intestinal barrier needs additional investigations.

Only a novel multi-omics approach, integrating all these factors in a new phenotype-“enterophenotype” model, may shed light on the complex pathophysiology of the autism disease by artificial intelligence-based algorithms to provide clinical decision support systems (CDSS) (Putignani et al., 2019). Surely, the small cohort sample size, especially for stratified groups, may represent a study limitation, and a further scale-up of our patients’ set will be performed to properly assess phenotype stratification.

In conclusion, for our patients cohort, the GM of the children with ASD was characterized by a well-differentiated gut microbial profile, featured by reduced richness and diversity as well as by specific composition and structure of bacterial communities and functions, dependently on host age and GI hallmarks.

## Data Availability Statement

The datasets presented in this study can be found in online repositories. The names of the repository/repositories and accession number(s) can be found below: PRJNA754695 (https://www.ncbi.nlm.nih.gov/bioproject).

## Ethics Statement

The studies involving human participants were reviewed and approved by Bambino Gesù Children’s Hospital (OPBG) Ethics Committees (1404_OPBG_2017 and 1113_OPBG_2016). Written informed consent to participate in this study was provided by the participants’ legal guardian/next of kin.

## Author Contributions

PV, MVR, SV, GI, AG, and LP contributed to the conceptualization. PV, AR, SGa, EL, and SGu contributed to the methodology. LP, PP, and SGa contributed to the validation. SA-N, PV, AR, FC, VG, and SGa contributed to the formal analysis. SGu, EL, GI, SV, and LP contributed to the investigation. LP contributed to the resources. PV, LP, and GI contributed to the data curation. PV, MVR, and LP wrote the original draft. PV, LP, and SGu contributed to the writing, reviewing, and editing the manuscript. AG, LP, AR, and SV contributed to the supervision. SV and LP contributed to the project administration and funding acquisition. All authors have read and agreed to the published version of the manuscript.

## Conflict of Interest

VG and SGa were employed by GenomeUp. The remaining authors declare that the research was conducted in the absence of any commercial or financial relationships that could be construed as a potential conflict of interest.

## Publisher’s Note

All claims expressed in this article are solely those of the authors and do not necessarily represent those of their affiliated organizations, or those of the publisher, the editors and the reviewers. Any product that may be evaluated in this article, or claim that may be made by its manufacturer, is not guaranteed or endorsed by the publisher.

## Footnotes

1
https://www.cdc.gov/nccdphp/dnpao/growthcharts/training/overview/page5_1.html

2
https://www.ncbi.nlm.nih.gov/bioproject/?term=PRJNA754695
