# Supplementary material for: Gut Microbiota Ecology and Inferred Functions in Children With ASD Compared to Neurotypical Subjects
Source: Front Microbiol. 2022 Jun 9;13:871086. doi: 10.3389/fmicb.2022.871086 (PMC9218677; doi:10.3389/fmicb.2022.871086)
Supplement: Supplementary file 1 [file Data_Sheet_1.docx]

**Supplementary Figures**

**
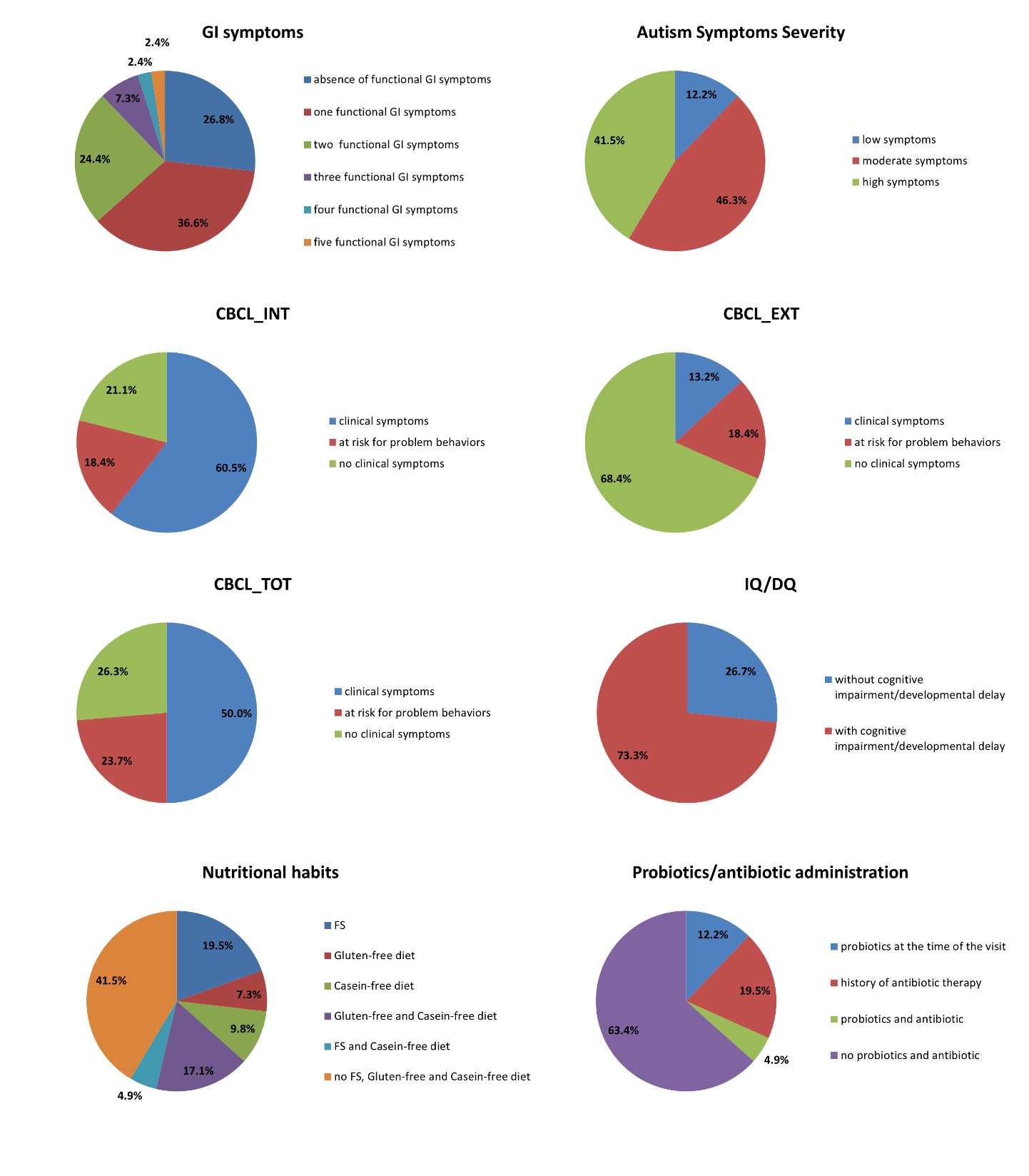
**

**Supplementary Figure 1.** Graphical representation of anamnestic and clinical features. Legend: CBCL, Child Behavior Checklist; CBCL_INT, CBCL_EXT, CBCL_TOT: Child Behavior Checklist Internalizing, Externalizing, and Total Problems Scales; FS: Food Selectivity.

**
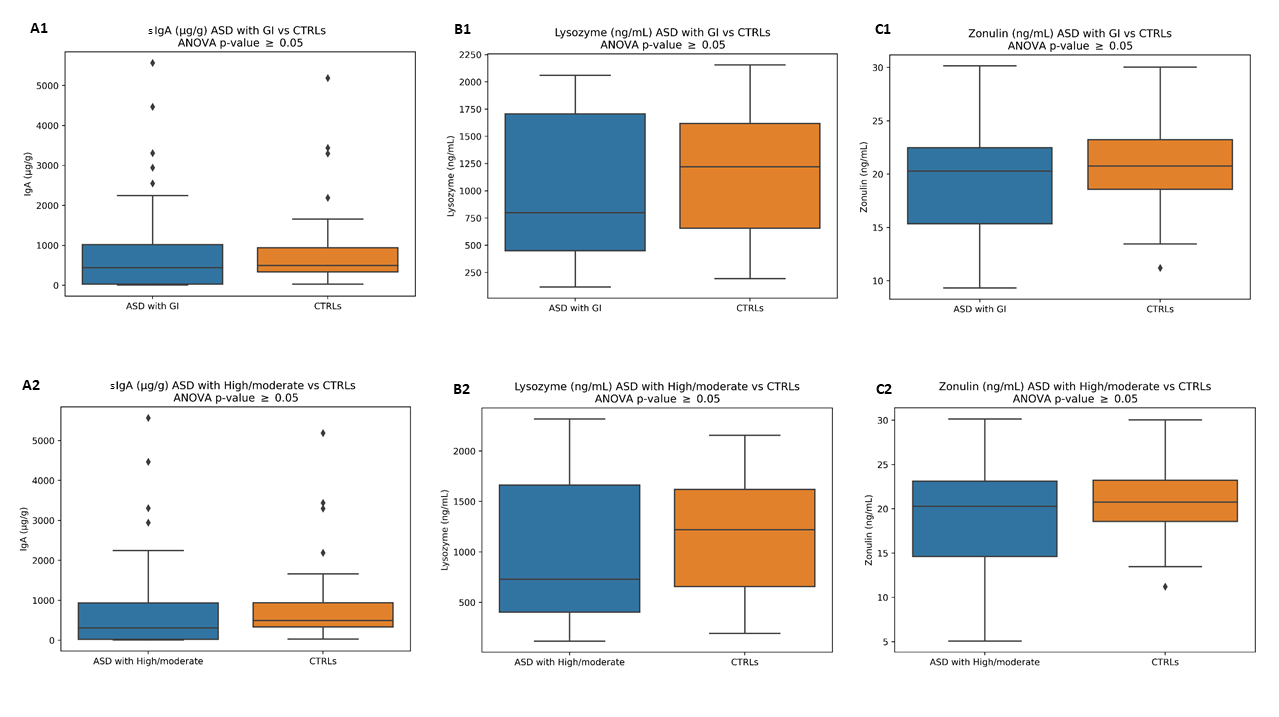
**

**Supplementary Figure 2.** Inflammation and intestinal permeability markers correlated with the presence of GI and high/moderate autism symptoms. **Panel A. A1**. sIgA comparison between ASD with GI versus CTRLs; **A2**. sIgA comparison between ASD with high/moderate versus CTRLs. **Panel B**. **B1** Lysozyme comparison between ASD with GI versus CTRLs; **B2**. Lysozyme comparison between ASD with high/moderate versus CTRLs. **Panel C**. **C1** Zonulin comparison between ASD with GI versus CTRLs; **C2**. Zonulin comparison between ASD with high/moderate versus CTRLs. The interquartile range is represented by the box and the line in the box is the median. The whiskers highest and lowest data points are reported, while the dots represent the outliers. All comparisons are not statistically significant (p value ≥0.05).


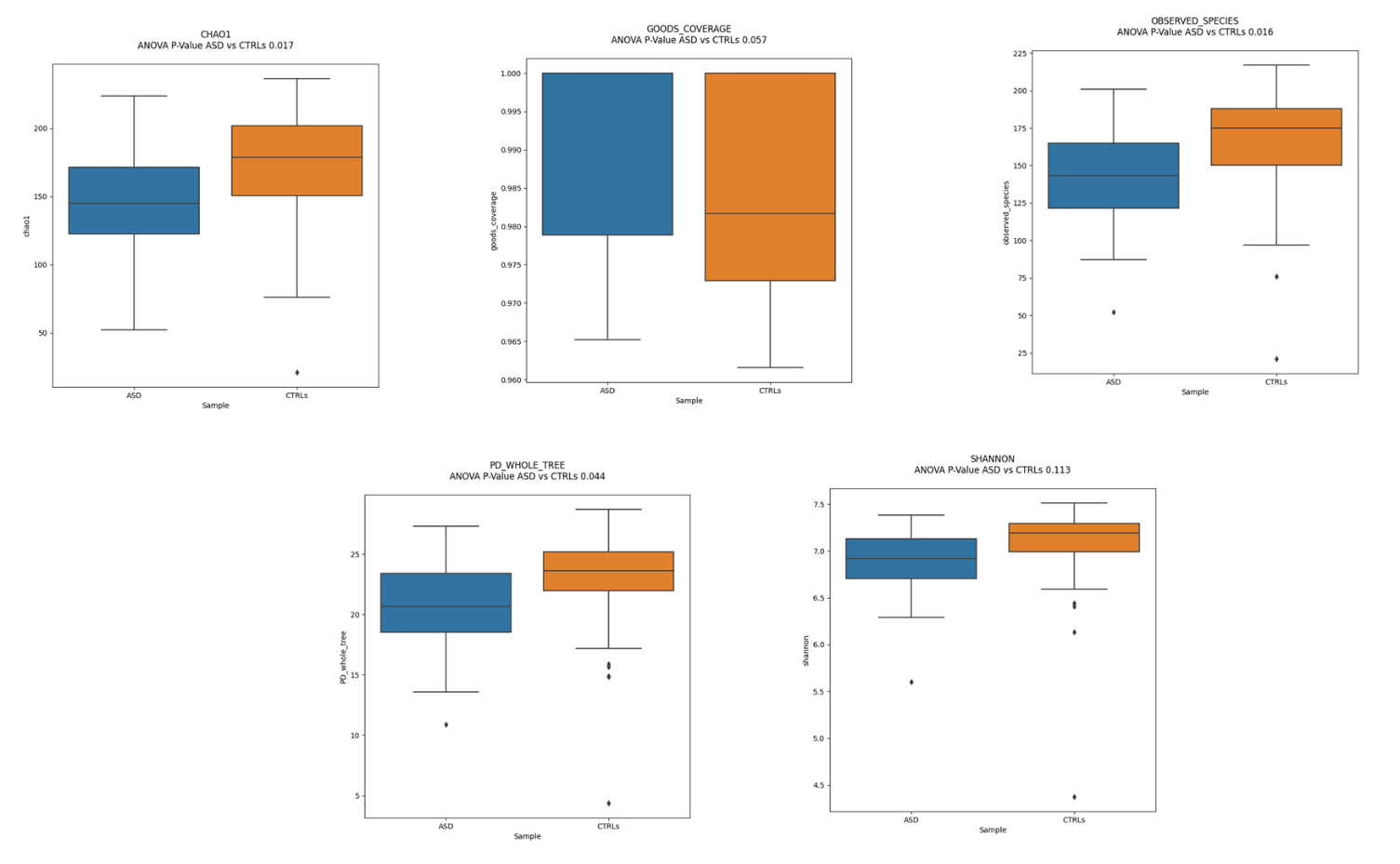


**Supplementary Figure 3**. Alpha diversity measured observed of CHAO1, Good Coverage, Observed species, Shannon and Simpson indices. Boxes represent the median, 25th and 75th percentile for CTRLs and ASD groups. P values are reported on the top of the figures.

**
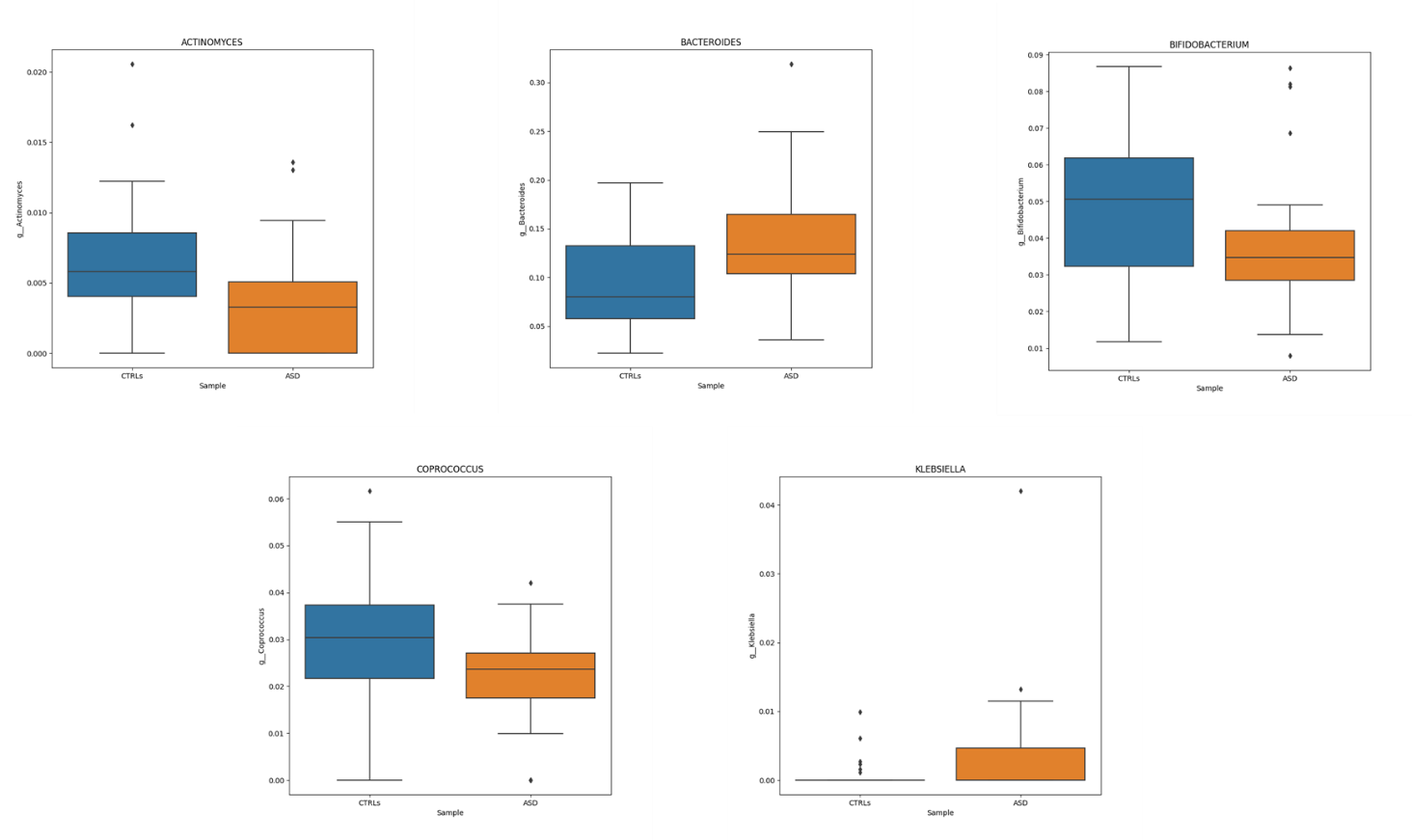
**

**Supplementary Figure 4.** Operational Taxonomic Unit (OTU) distribution (genus level) of the GM in ASD and CTRLs after Mann-Whitney U test filtering. The interquartile range is represented by the box and the line in the box represents the median. The whiskers highest and lowest data points are reported, while the dots represent the outliers. Only statistically significant OTUs (Mann-Whitney U p value FDR ≤ 0.05) are represented.

**
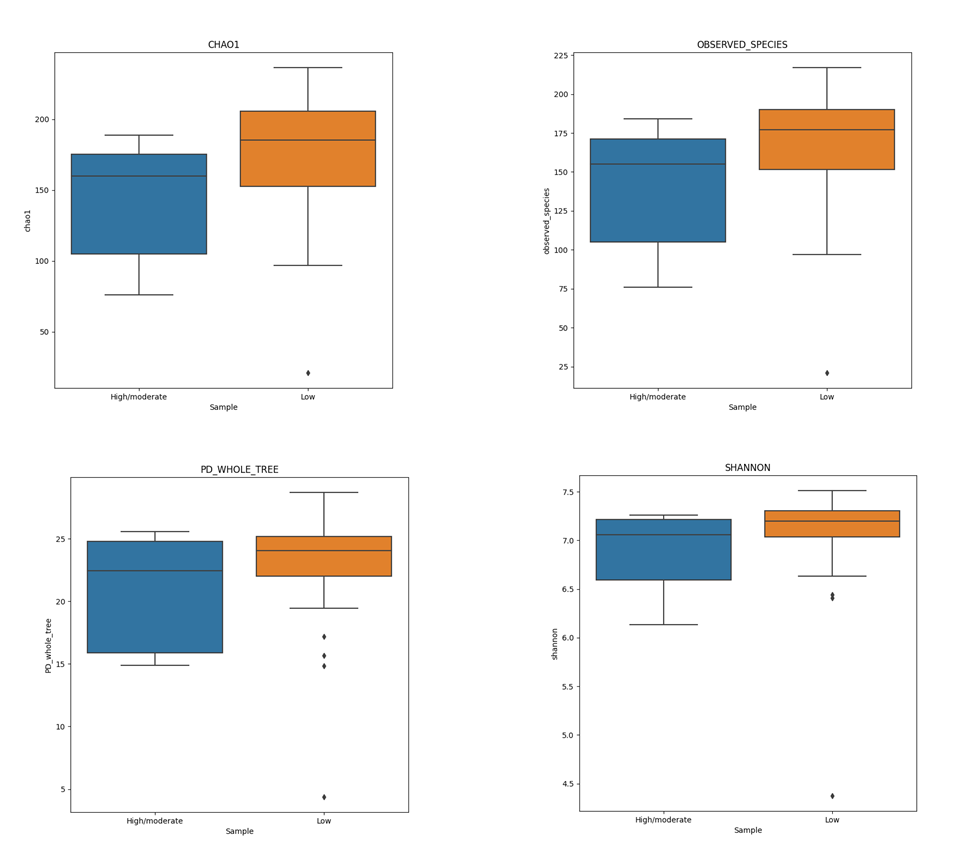
**

**Supplementary Figure 5.** Alpha diversity measured observed of CHAO1, Observed species, PD_Whole_Tree and Shannon. Boxes represent the median, 25th and 75th percentile for patients with ASD according to autism symptoms severity, grouped in high/moderate versus low. All comparisons are not statistically significant (p value ≥0.05).


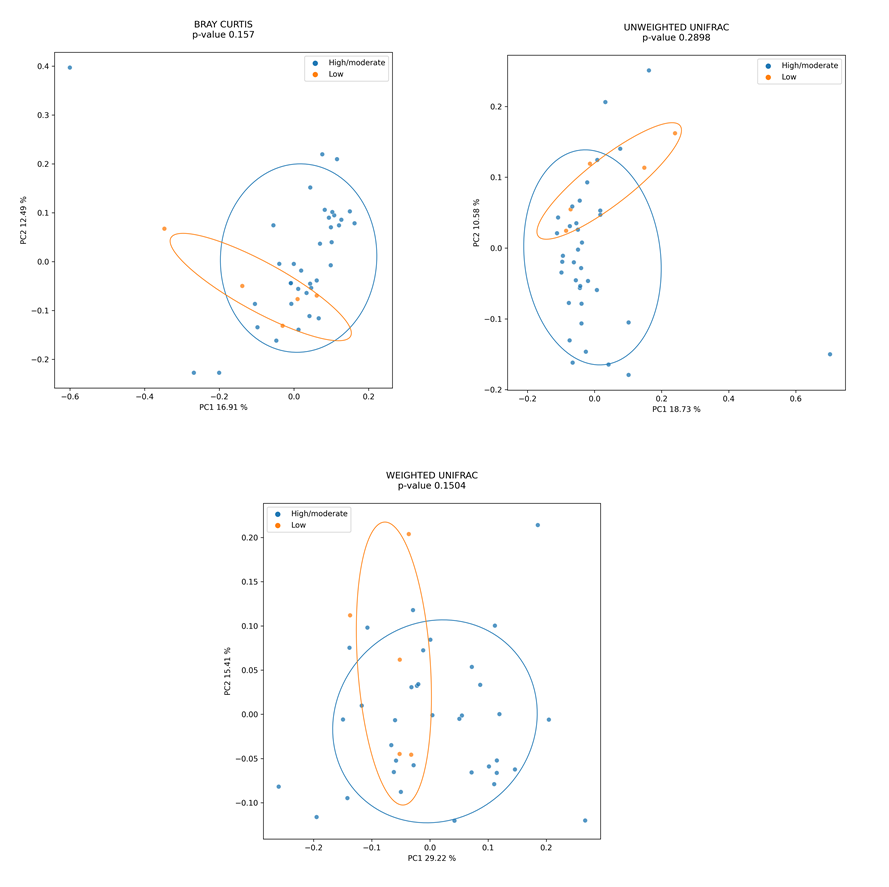


**Supplementary Figure 6**. Beta-diversity ecological analysis. Principal Coordinate Analysis (PCoA) plots shows Bray Curtis, Weighted and unweighted UniFrac algorithms applied on patients with ASD according to autism symptoms severity, grouped into high/moderate versus low. P values are reported on the top of the figures.


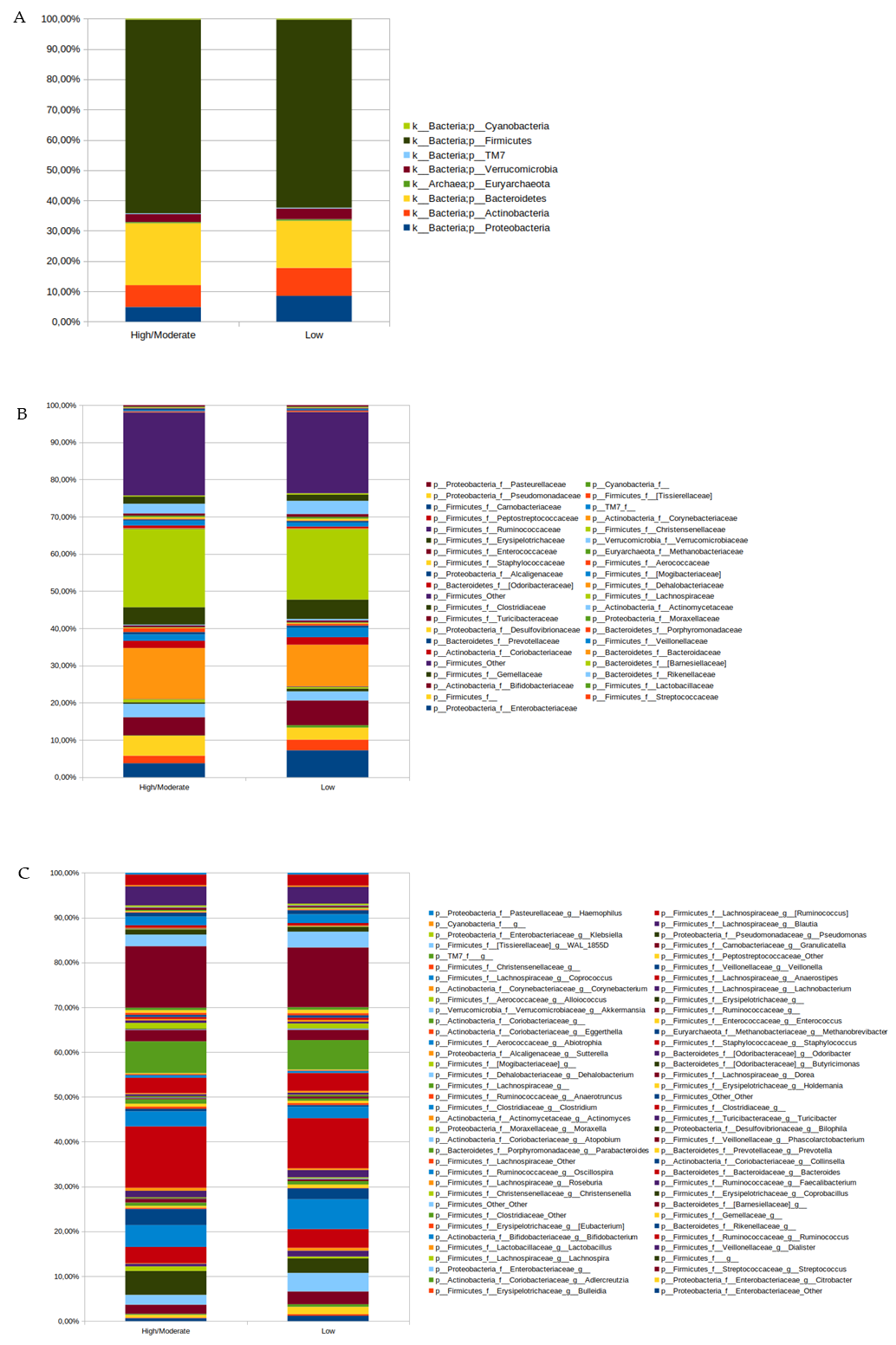


**Supplementary Figure 7**. OTUs distribution performed by Mann-Whitney U-test applied on patients with ASD according to autism symptoms severity, grouped into high/moderate versus low. **Panel A**. Phylum **panel B**, family level and **panel C**, genus level. All comparison are not statistically significant (p value ≥0.05).


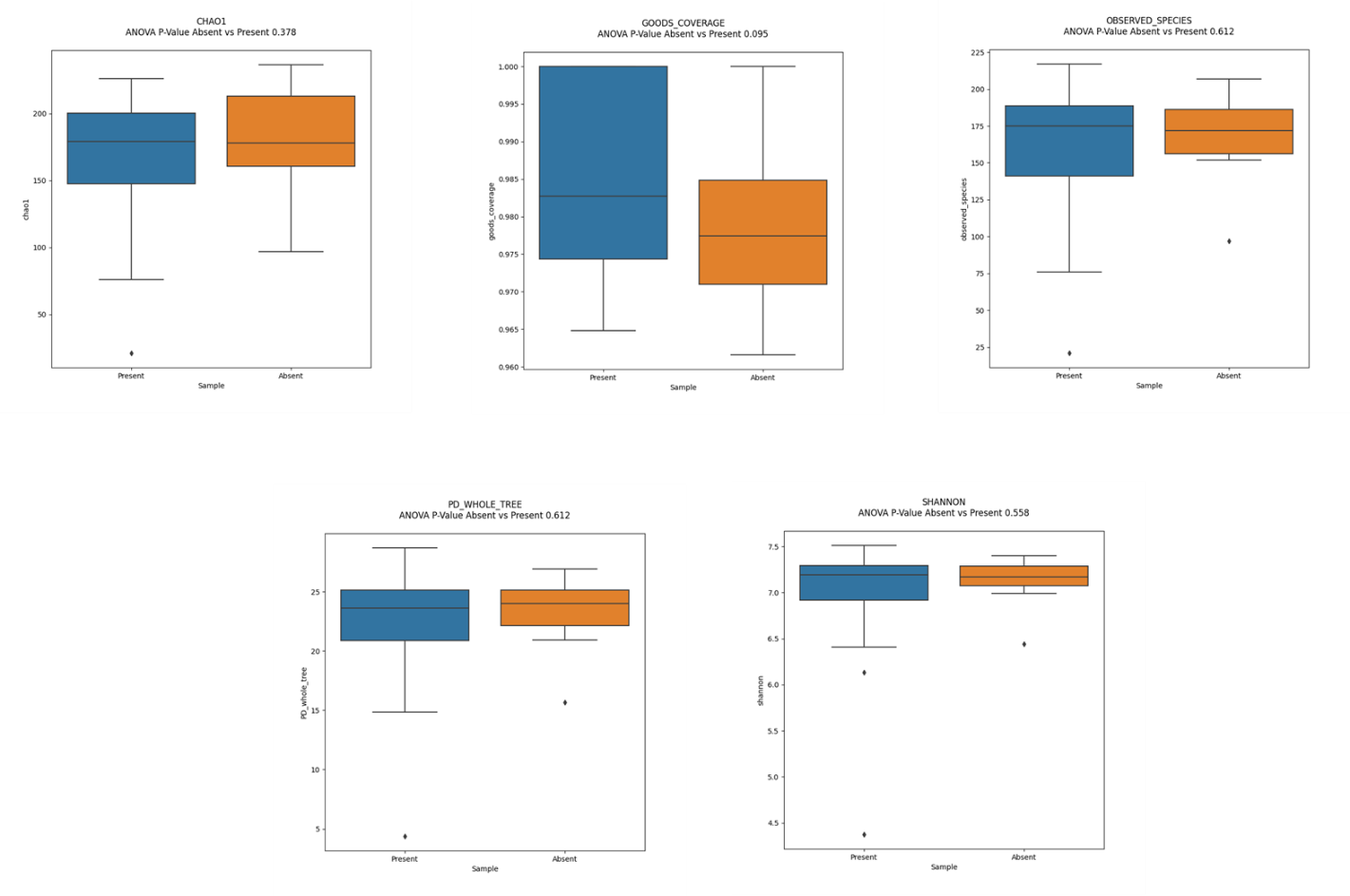


**Supplementary Figure 8.** Alpha diversity measured observed of CHAO1, Observed species, Goods coverage, Oserved species, PD_Whole_Tree and Shannon. Boxes represent the median, 25th and 75th percentile for patients with ASD according only to food selectivity, grouped in No-Food Selectivity (absent) versus Food Selectivity (present) All comparisons are not statistically significant (p value ≥0.05).


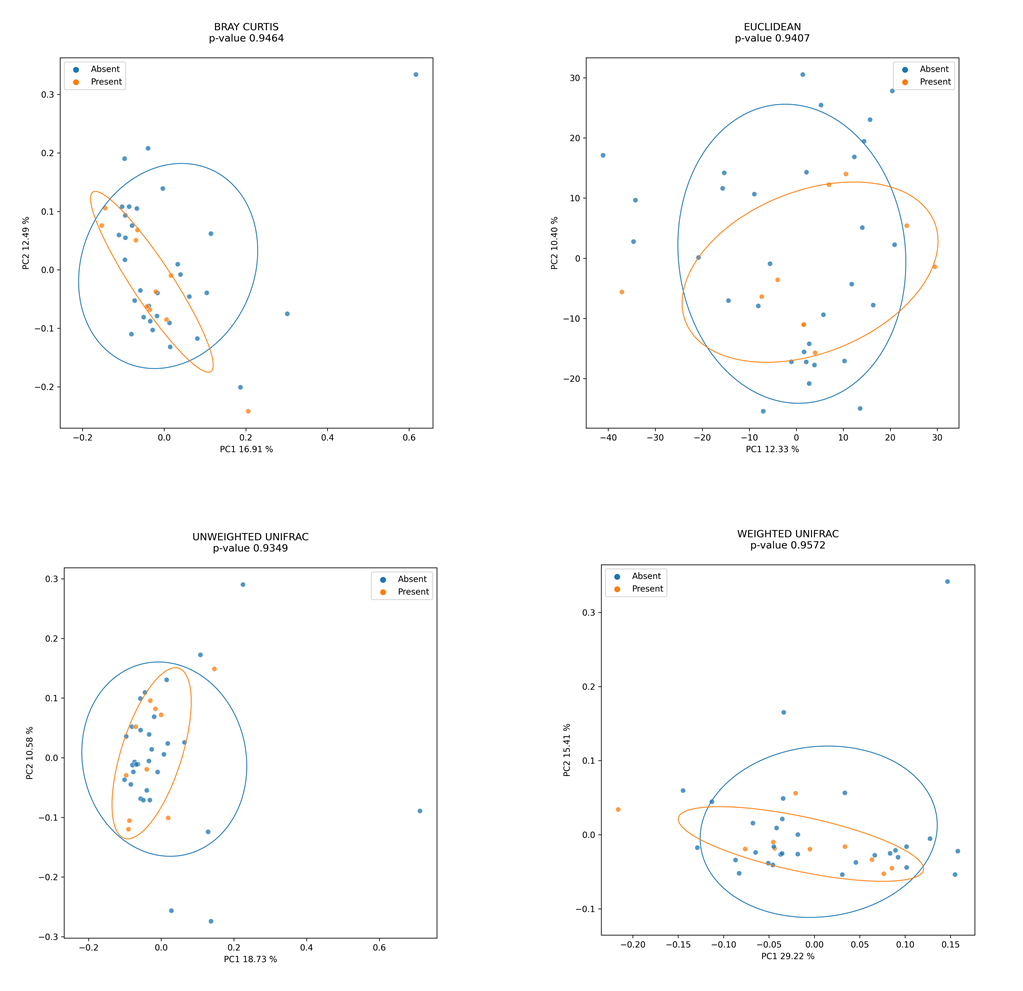


**Supplementary Figure9**. Beta-diversity ecological analysis. Principal Coordinate Analysis (PCoA) plots shows Bray Curtis, Euclidean, Weighted and unweighted UniFrac algorithms applied on patients with ASD according to only to food selectivity, grouped in No-Food Selectivity (absent) versus Food Selectivity (present). P values are reported on the top of the figures.


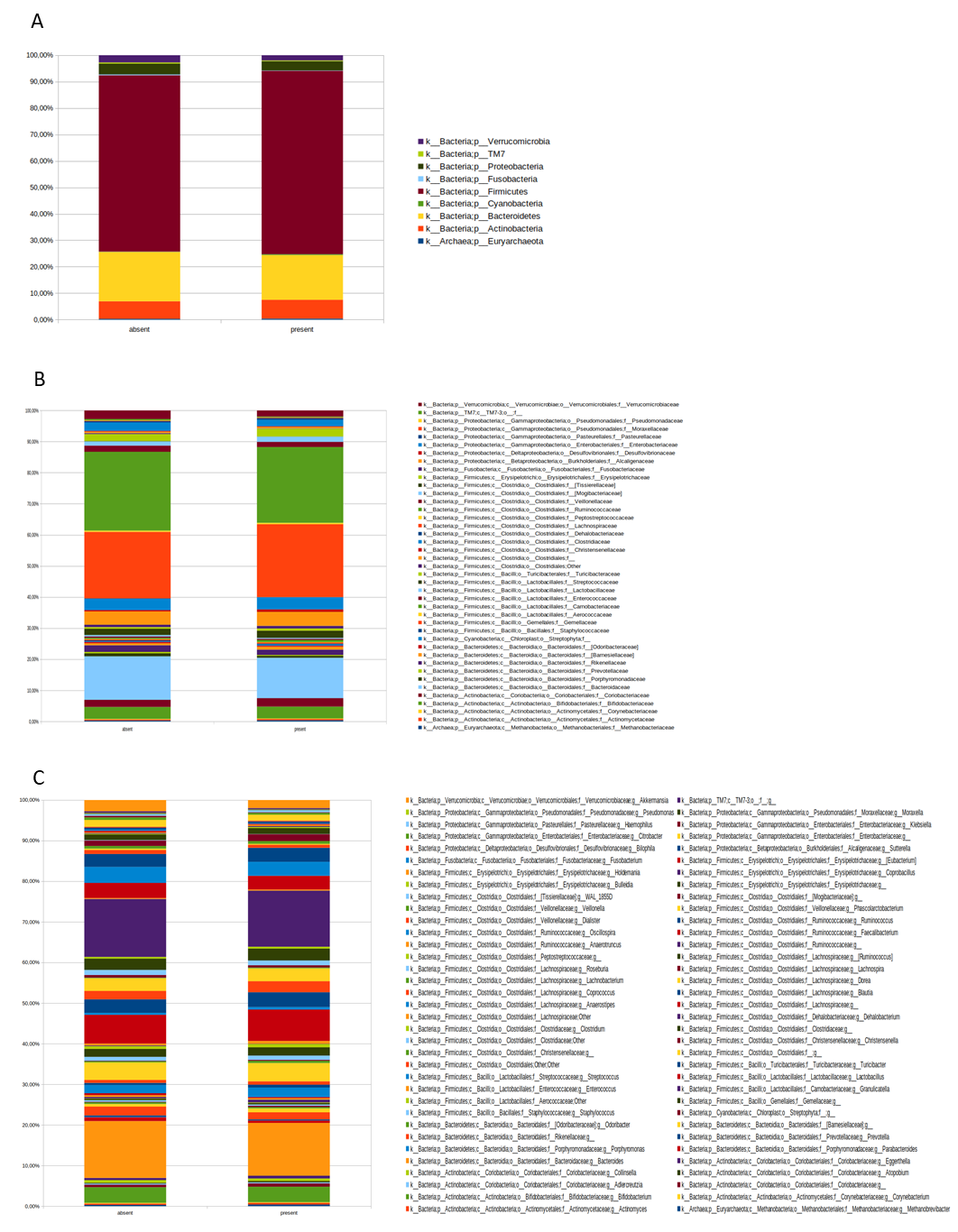


**Supplementary Figure 10**. OTUs distribution performed by Mann-Whitney U-test applied on patients with ASD according to only to food selectivity, grouped in no-Food Selectivity (absent) versus Food Selectivity (present). **Panel A**. Phylum **panel B**, family level and **panel C**, genus level. All comparison are not statistically significant (p value ≥0.05).

**
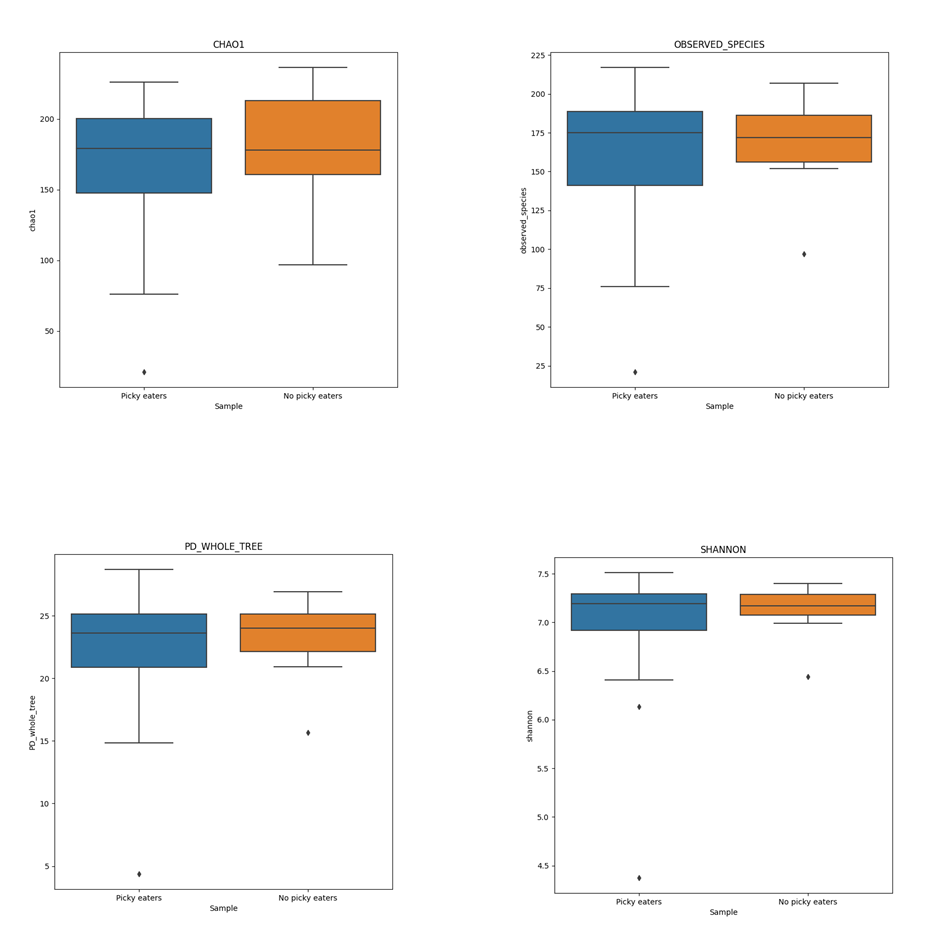
**

**Supplementary Figure 11.** Alpha diversity measured observed of CHAO1, Observed species, PD_Whole_Tree and Shannon. Boxes represent the median, 25th and 75th percentile for patients with ASD grouped for picky eaters (food selectivity, gluten and casein free diet) and no-picky eaters (NO food selectivity, gluten and casein free diet). All comparison are not statistically significant (p value ≥0.05).


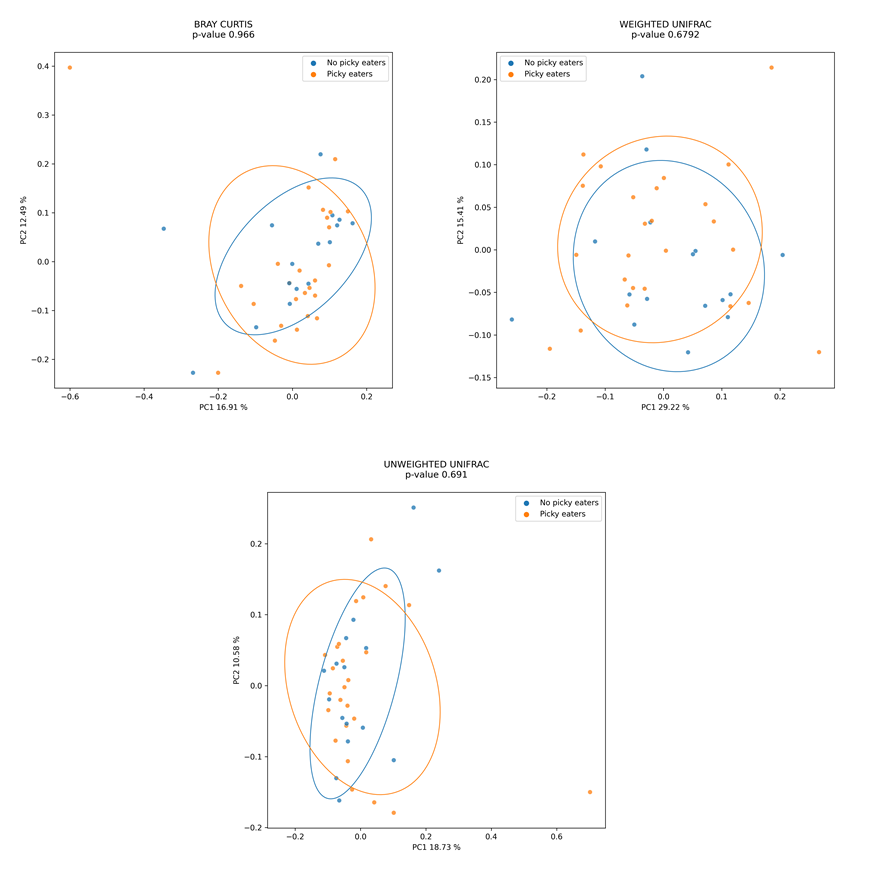


**Supplementary Figure 12.** Beta-diversity ecological analysis. Principal Coordinate Analysis (PCoA) plots shows Bray Curtis, Weighted and unweighted UniFrac algorithms applied on patients with ASD grouped for picky eaters (food selectivity, gluten and casein free diet) versus no- picky eaters (NO food selectivity, gluten and casein free diet). P values are reported on the top of the figures.

**
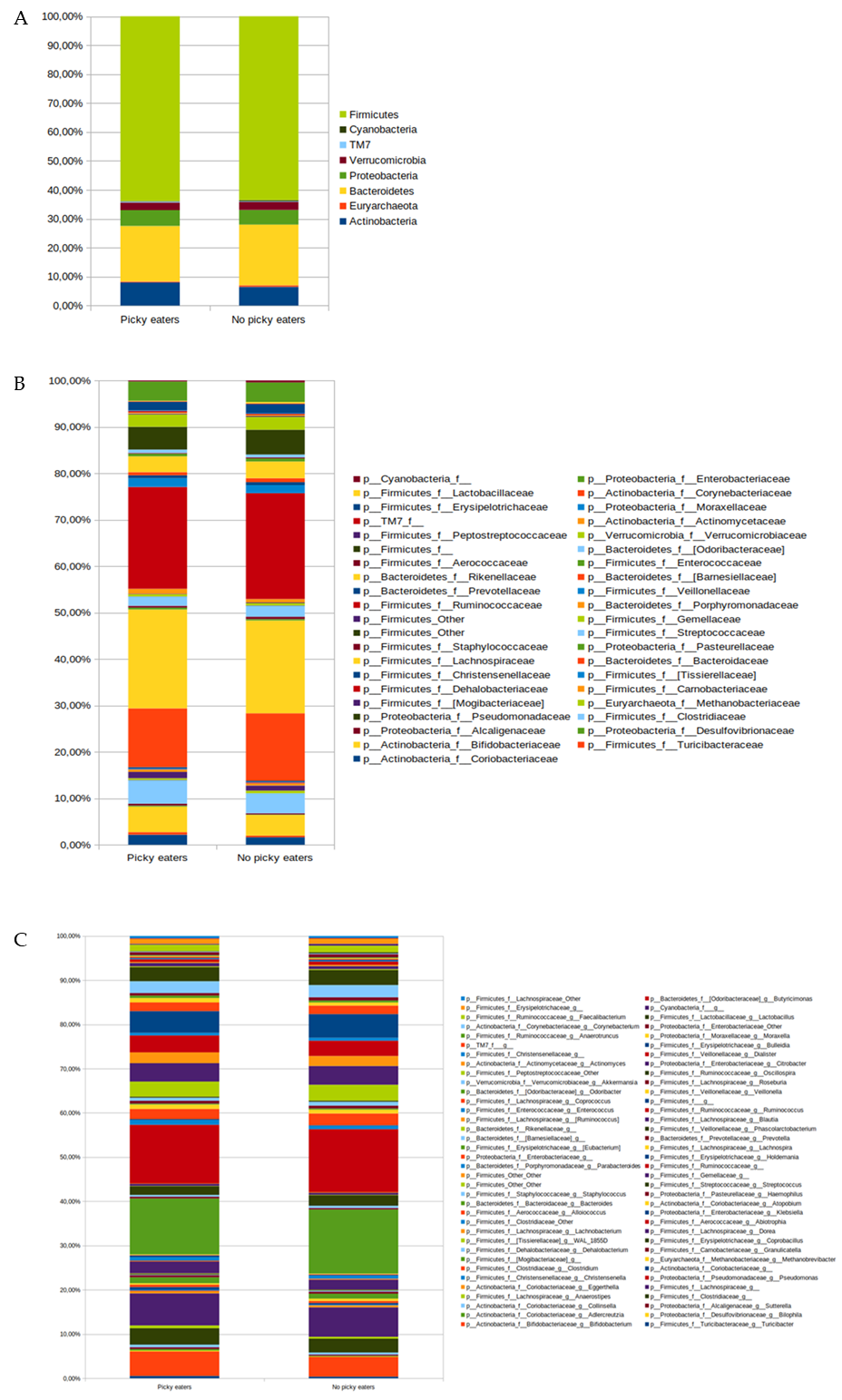
**

**Supplementary Figure 13**. OTUs distribution performed by Mann-Whitney U-test applied on patients with ASD grouped for picky eaters (food selectivity, gluten and casein free diet) and versus no-picky eaters (NO food selectivity, gluten and casein free diet). **Panel A**. Phylum; **panel B**, family and **panel C**, genus level. All comparison are not statistically significant (p value ≥0.05).


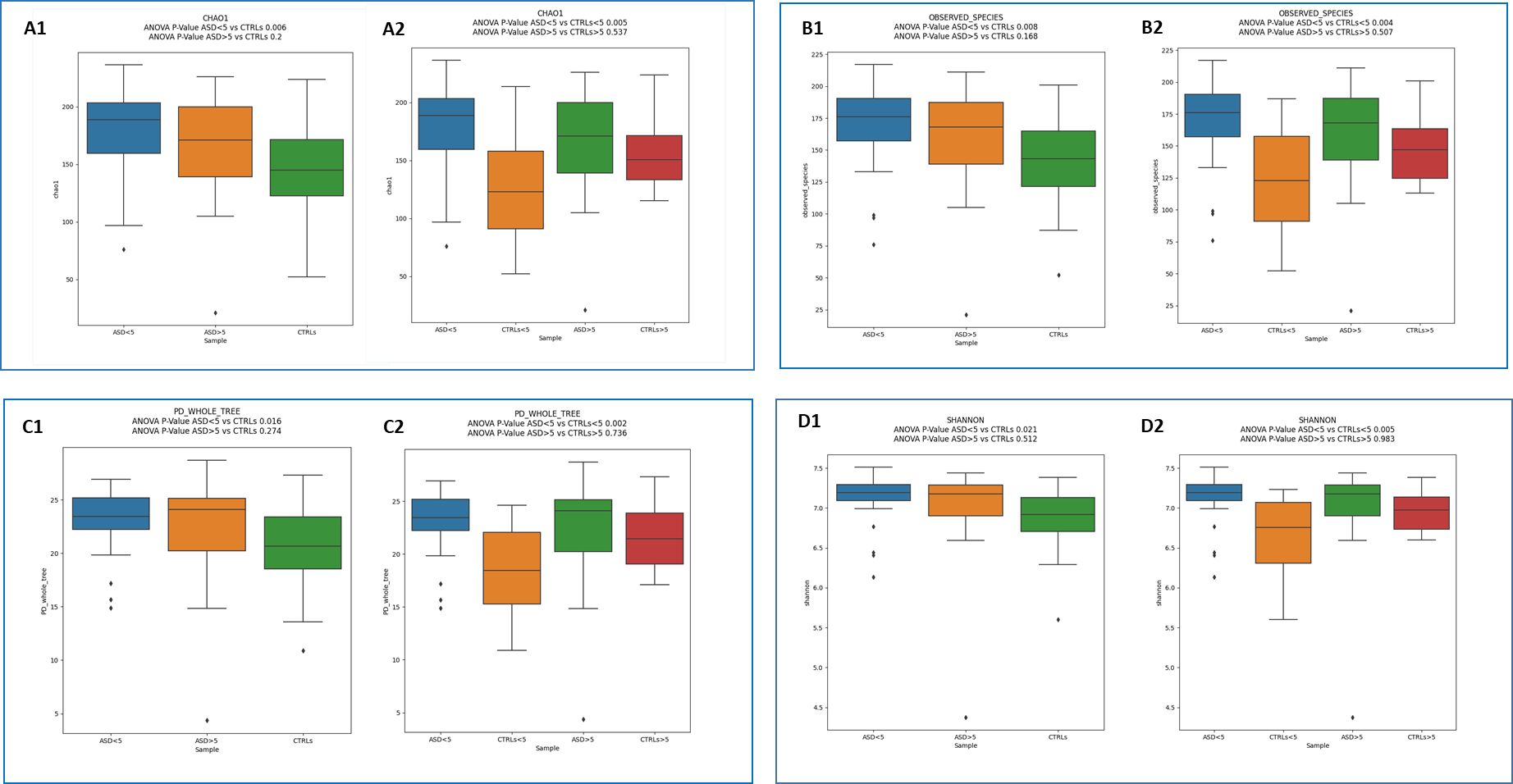


**Supplementary Figure 14.** Alpha diversity measured by CHAO1, Observed species, PD_Whole_Tree and Shannon algorithms. Boxes represent the median, 25th and 75th percentile for CTRLs, and patients with ASD grouped for age (<0-5 years old or ≥ 5 years old). **Panels** **A1, B1, C1, D1**. Pairwaise comparison between entire group of CTRLs versus i) ASD < 5 years old; ii) ASD≥5 years old. **Panels** **A2, B2, C2, D2.** Pairwaise comparison between i) ASD < 5 versus CTRLs <5; ii) ASD≥5 versus CTRLs ≥5 years old. P values are reported on the top of panels.


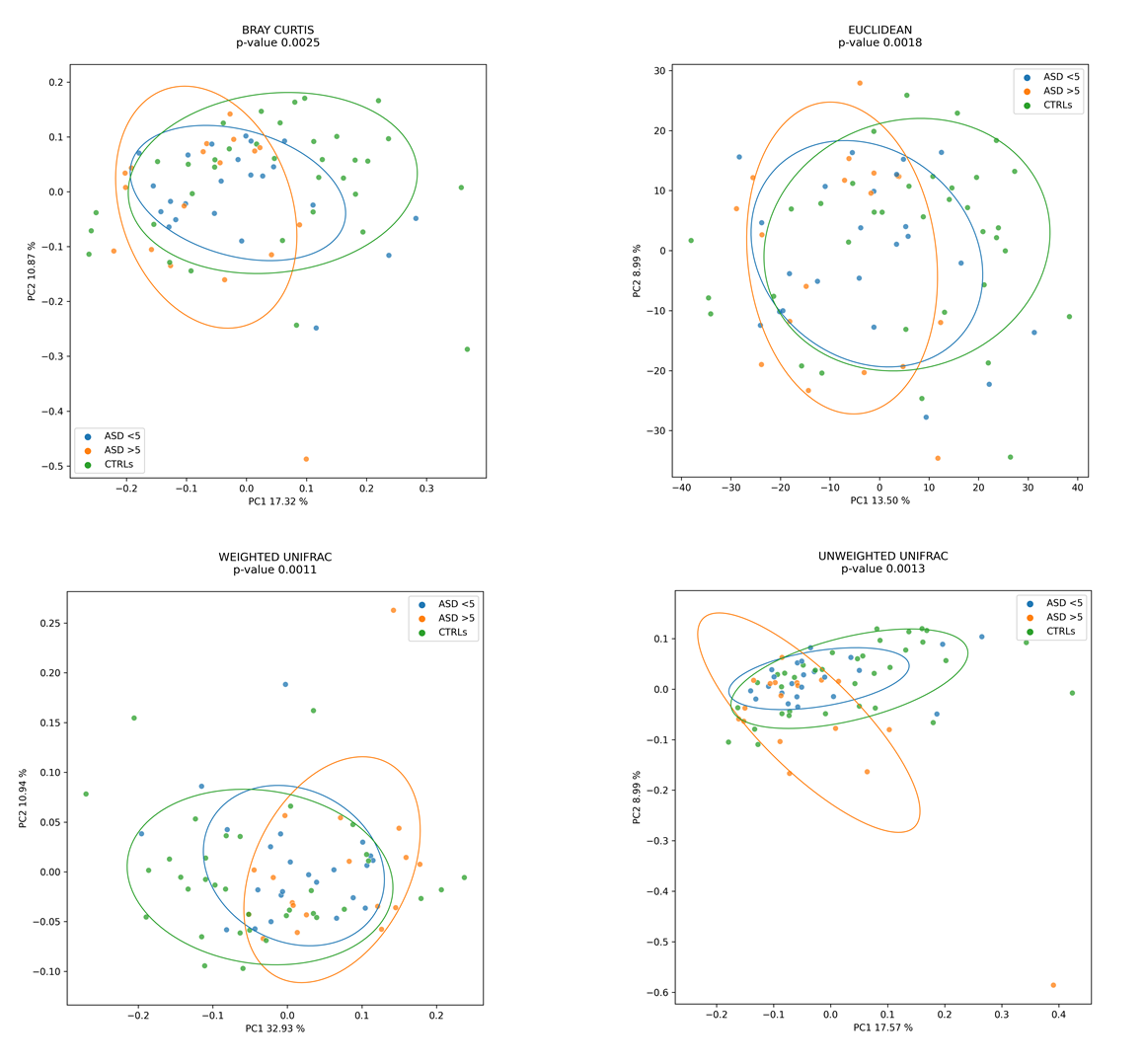


**Supplementary Figure 15.** Beta-diversity ecological analyses. Principal Coordinate Analysis (PCoA) plots are based on Bray Curtis, Euclidean distance, Weighted and Unweighted UniFrac algorithms applied on CTRLs and patients with ASD grouped for age (<0-5 years old or ≥ 5 years old). P values are reported on the top of the figures.


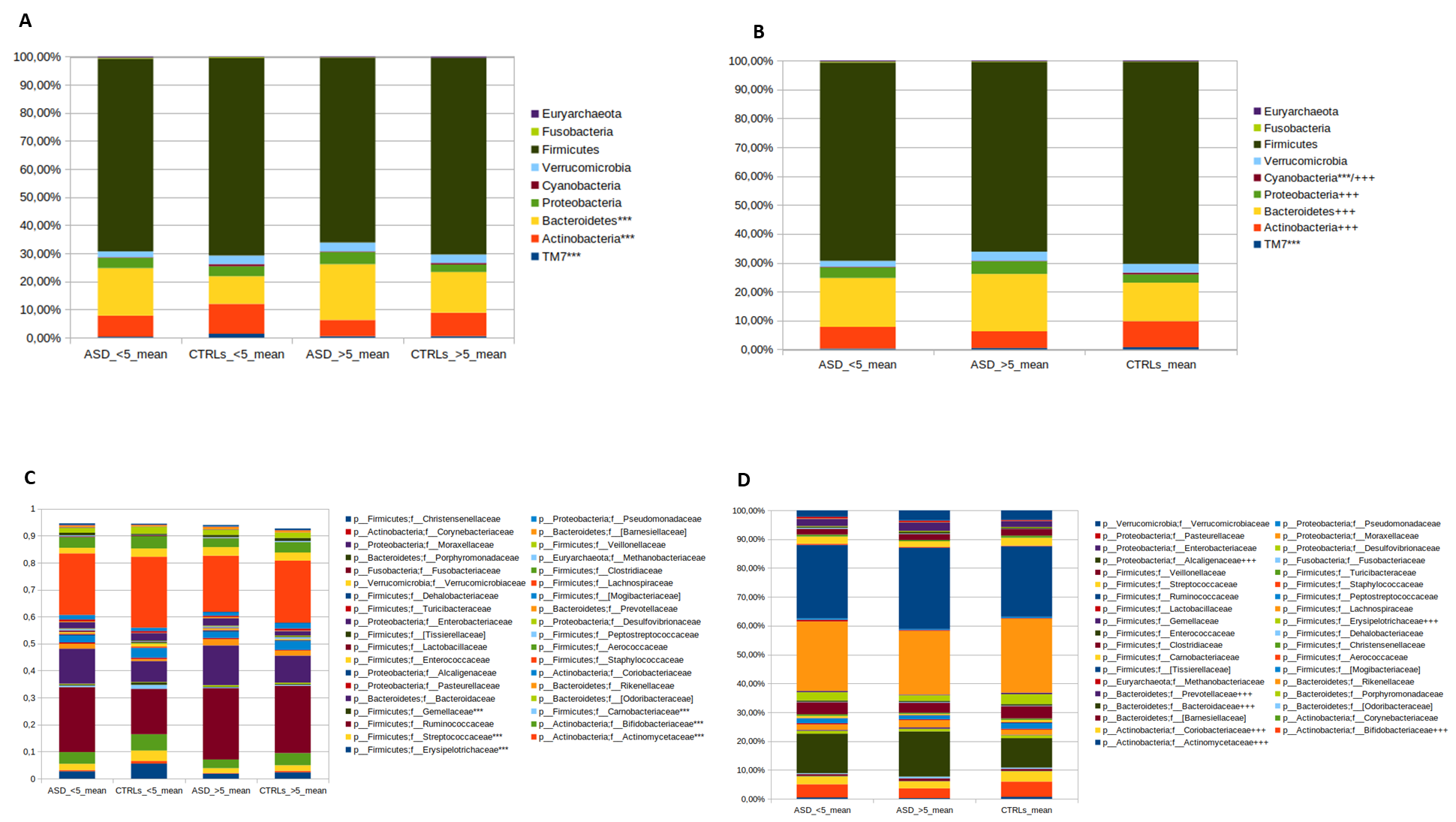


**Supplementary Figure 16.** OTUs distribution performed by Kruskal-Wallis and Mann-Whitney U-test applied to the all and coupled-comparison, respectively, between CTRLs and patients with ASD both grouped for age (0-5 years old or ≥ 5 years old) **Panel A**. Kruskal Wallis test between age groups at phylum level. **panel B**, Mann-Whitney test applied to the entire group of CTRLs versus i) ASD < 5; ii) ASD≥5 years old at phylum level; **panel C**. Kruskal Wallis test amongst age groups at family level; **panel D.** Mann-Whitney U-test applied on the entire group of CTRLs versus i) ASD < 5; ii) ASD≥5 years old at family level. **Legend.** **Panel A**.******* FDR<0.05; **Panel B**. *******FDR<0.05 for ASD <5 versus CTRL and ^+++^FDR<0.05 for ASD≥5 versus CTRL; **Panel C**. *** FDR<0.05; **Panel D**. *******FDR<0.05 for ASD <5 versus CTRL and ^+++^FDR<0.05 for ASD≥5 versus CTRL.

**
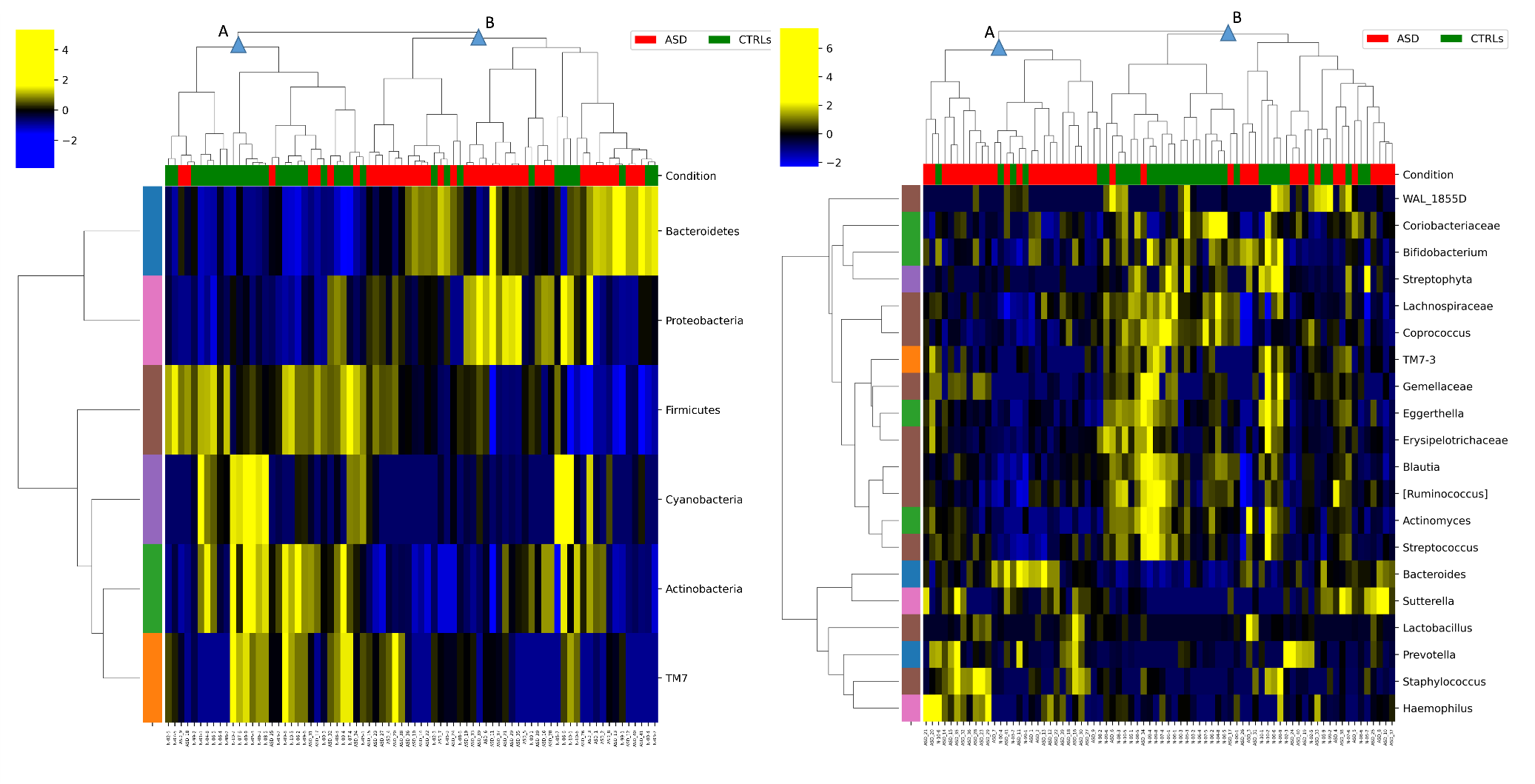
**

**Supplementary Figure 17.** Graphical representation of hierarchical analysis of global OTUs distribution for ASD and CTRLs subjects filtered by a t-test between classes with p value <0.05. Panel A. Level L2 (phylum level) and Panel B. Level L6 (genus). In the heatmap, the hierarchical complete-linkage dendrogram is based on the OTUs Pearson’s correlation coefficient. The color scale characterizes the Z- score for each variable: yellow, high level; blue, low level. The column bar colors represent the subject condition category (red, ASD; green, CTRLs).

**
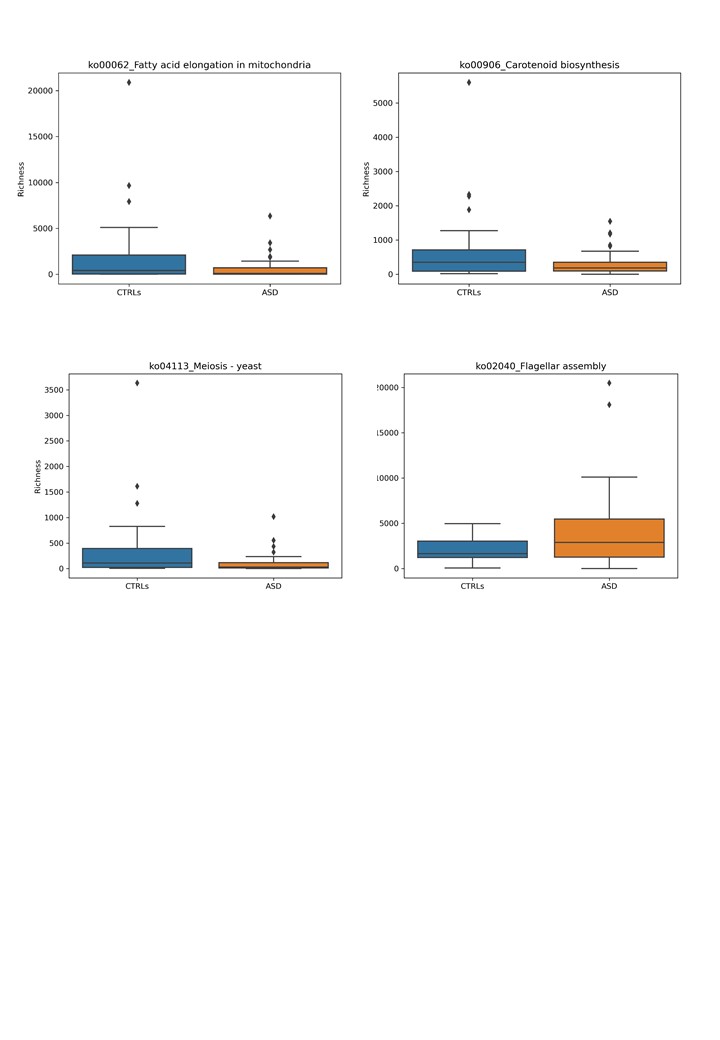
**

**Supplementary Figure 18**. Main ko pathways classifying the 73% of ASDs patients versus CTRLs computed by machine learning and selected by t-test (p value ≤ 0.05).
