# Supplementary material for: Gut Microbiota Ecology and Inferred Functions in Children With ASD Compared to Neurotypical Subjects
Source: Front Microbiol. 2022 Jun 9;13:871086. doi: 10.3389/fmicb.2022.871086 (PMC9218677; doi:10.3389/fmicb.2022.871086)
Supplement: Supplementary file 5 [file Table_4.DOCX]

**Supplementary Tables**

**Supplementary Table 4.** KEEG pathways derived from the comparison between to CTRLs versus ASD with GI symptoms

| **KEGG pathways** | **Class*** | **Subclass** | **Group** | **KEGG pathways** | **Class** | **Subclass** | **Group** |
| --- | --- | --- | --- | --- | --- | --- | --- |
| Chloroalkane and chloroalkene degradation | 1 | Carbohydrate metabolism | CTRLs | Starch and sucrose metabolism | 1 | Carbohydrate metabolism | ASD with GI |

*****Class, 1. Metabolism.

**Supplementary Table 5.** KEEG pathways derived from the comparison between CTRLs versus ASD without GI symptoms

| **KEGG pathways** | **Class*** | **Subclass** | **Group** |
| --- | --- | --- | --- |
| Fluorobenzoate degradation | 1 | Nucleotide metabolism | ASD wihout GI |
| Vasopressin-regulated water reabsorption | 2 | Signal transduction |  |

*****Class, 1. Metabolism; 2. Environmental Information Processing.

**Supplementary Table 6.** KEEG pathways derived from the comparison between ASD with GI symptoms versus ASD without GI symptoms

| **KEGG pathways** | **Class*** | **Subclass** | **Group** | **KEGG pathways** | **Class** | **Subclass** | | **Group** |
| --- | --- | --- | --- | --- | --- | --- | --- | --- |
| mRNA surveillance pathway | 1 | Transcription | ASD with GI | Fluorobenzoate degradation | 2 | Nucleotide metabolism | ASD without GI | |

*****Class, 1. Genetic Information Processing; 2. Metabolism

**Supplementary Table 7.** Classification models applied to discriminate ASD and CTRLs groups on gut microbiota composition at genus level.

| **Model** | **Score** | **Score ASD** |  | **Score CTRLs** |
| --- | --- | --- | --- | --- |
| Dummy Classifier | 0.500 | 1.000 |  | 0.000 |
| Logistic Regression | 0.731 | 0.917 |  | 0.545 |
| SGD Classifier | 0.735 | 0.833 |  | 0.636 |
| Logistic Regression CV | 0.686 | 0.917 |  | 0.455 |
| Hist Gradient Boosting Classifier | 0.689 | 0.833 |  | 0.545 |
| Random Forest Classifier | 0.689 | 0.833 |  | 0.545 |
| Extra Trees Classifier | 0.686 | 0.917 |  | 0.455 |
| Gradient Boosting Classifier | 0.689 | 0.833 |  | 0.545 |
| Bagging Classifier | 0.644 | 0.833 |  | 0.455 |
| Ada Boost Classifier | 0.689 | 0.833 |  | 0.545 |
| XGB Classifier | 0.644 | 0.833 |  | 0.455 |
| XGBRF Classifier | 0.648 | 0.750 |  | 0.545 |
| MLP Classifier | 0.731 | 0.917 |  | 0.545 |
| Linear SVC | 0.731 | 0.917 |  | 0.545 |
| SVC | 0.731 | 0.917 |  | 0.545 |
| Gaussian NB | 0.773 | 1.000 |  | 0.545 |
| Decision Tree Classifier | 0.686 | 0.917 |  | 0.455 |
| Quadratic Discriminant Analysis | **0.867** | 0.917 |  | 0.818 |
| K Neighbors Classifier | 0.640 | 0.917 |  | 0.364 |
| Gaussian Process Classifier | **0.777** | 0.917 |  | 0.636 |
